# Can low-dose intravenous immunoglobulin be an alternative to high-dose intravenous immunoglobulin in the treatment of children with newly diagnosed immune thrombocytopenia: a systematic review and meta-analysis

**DOI:** 10.1186/s12887-024-04677-3

**Published:** 2024-03-21

**Authors:** Xiangge Ren, Miaomiao Zhang, Xiaohan Zhang, Peidong Zhao, Wensheng Zhai

**Affiliations:** 1 Department of Pediatrics, Children’s Purpura and Nephropathy Center, The first affiliated hospital of Henan University of Chinese Medicine, No.19, Renmin Road, Jinshui District, Zhengzhou, Henan China; 2 College of Pediatrics, Henan University of Chinese Medicine, No.156, Jinshui East Road, Jinshui District, Zhengzhou, Henan China

**Keywords:** Newly diagnosed immune thrombocytopenia, Intravenous immunoglobulin, Children, Dose, Meta-analysis

## Abstract

Intravenous immunoglobulin (IVIg) is a first-line treatment for children with newly diagnosed immune thrombocytopenia (ITP). Higher doses of IVIg are associated with a more insupportable financial burden to pediatric patients’ families and may produce more adverse reactions. Whether low-dose IVIg (LD-IVIg) can replace high-dose IVIg (HD-IVIg) has yet to be established. We conducted a comprehensive literature search from the establishment of the database to May 1, 2023, and eventually included 22 RCTs and 3 cohort studies compared different dosages of IVIg. A total of 1989 patients were included, with 991 patients in the LD-IVIg group and 998 patients in the HD-IVIg group. Our results showed no significant differences between the two groups in the effective rate (LD-IVIg: 91% vs. HD-IVIg: 93%; RR: 0.99; 95%CI: 0.96–1.02) and the durable remission rate (LD-IVIg: 65% vs. HD-IVIg: 67%; RR: 0.97; 95%CI: 0.89–1.07). Similar results were also found in the time of platelet counts (PC) starting to rise (MD: 0.01, 95%CI: -0.06–0.09), rising to normal (MD: 0.16, 95%CI: -0.03–0.35), and achieving hemostasis (MD: 0.11, 95%CI: -0.02–0.23) between the two groups. Subgroup analysis showed the effective rate of 0.6 g/kg was equal to 1 g/kg subgroup (91%) but higher than 0.8 g/kg subgroup (82%), and a combination with glucocorticoid may contribute to effect enhancement (combined with glucocorticoid: 91% vs. IVIg alone: 86%) whether combined with dexamethasone (92%) or methylprednisolone (91%). Besides, the incidence rate of adverse reactions in the LD-IVIg group (3%) was significantly lower than the HD-IVIg group (6%) (RR: 0.61; 95%CI: 0.38–0.98). So low-dose IVIg (≤ 1 g/kg) is effective, safe, and economical, which can replace high-dose IVIg (2 g/kg) as an initial treatment. This systematic review was registered in PROSPERO (CRD42022384604).

**Supplementary Information:**

The online version contains supplementary material available at 10.1186/s12887-024-04677-3.

## Introduction

Immune thrombocytopenia (ITP) is an acquired autoimmune hemorrhagic disease characterized by decreased platelet counts (< 100 × 10^9^/L) and coagulation function due to immune-mediated platelet destruction and impaired platelet production [1, 2]. It is the most common cause of thrombocytopenia in childhood, with an estimated incidence of approximately 1.9–6.4 per 100,000 children per year [3].

New onset of epistaxis, gingival bleeding, petechiae, and ecchymoses is typical in pediatric patients at diagnosis [4], many of whom will experience spontaneous resolution and will not require medical treatment [5, 6]. However, about 20.2% of children may develop severe bleeding [7], adversely affecting the health-related quality of life and need medical treatment [8].

Currently, the treatment of ITP is not strictly regimented. First-line therapy for children with newly diagnosed ITP typically consists of glucocorticoids, intravenous immunoglobulin (IVIg), or a combination of both [9]. IVIg is a blood product prepared from the serum of many healthy donors, containing microbial antigens, autoantigens, and anti-idiotype antibodies [10]. Studies about the pharmacological mechanism have indicated that IVIg inhibits the Fc-mediated phagocytosis of antibody-coated platelets by the reticuloendothelial system [4, 11, 12], meanwhile encouraging the development or activation of T cells and regulating the function of B cells [13, 14], thereby protecting platelets and suppressing autoimmune responses. Recently, Schmugge et al. identified that IVIg may have the ability to improve thrombin-induced platelet activation and enhance thrombin generation in a prospective observational study of 23 children with newly diagnosed ITP, indicating that besides increasing platelet counts, IVIg treatment helps to counteract diminished platelet function and coagulation [15].

However, the costs of IVIg are high, which is hardly bearable for many families. A study showed the mean hospitalization cost for ITP children treated with IVIg was $6275 [16]. Since the administration of IVIg requires an inpatient admission, the cost increase is most pronounced for those who need high-dose IVIg. Furthermore, high-dose use was considered as one of the main risk factors for undesirable IVIg-associated adverse events such as flu-like symptoms, dermatological adverse effects, thrombotic events, aseptic meningitis, hemolysis, and renal failure [17].

To alleviate patients’ financial pressure and ensure efficacy and safety, how to rationally reduce the dosage of IVIg is always a matter of clinical concern, especially in some developing countries. In China, IVIg is administered chiefly at a total dose of 2 g/kg distributed over 2–5 days [18]. Although several studies have shown that reducing the dosage of IVIg may also be effective and can be used for ITP in newly treated children, there is still some controversy on the optimal low-dose regimen.

Thus, we performed a comprehensive analysis to evaluate both long and short-term efficacy of low-dose IVIg and to compare the effect of different low-dose regimens and combination with glucocorticoids, which may provide enough information for healthcare providers to choose the most appropriate medication regimen when treating children with newly diagnosed ITP.

## Methods

### Protocol and registration

This systematic review was reported following Preferred Reporting Items for Systematic Reviews and Meta-analyses (PRISMA) [19] and registered in PROSPERO (CRD42022384604).

### Literature search and selection

We conducted an extensive search strategy to retrieve all eligible literature published from the establishment of the database to May 1, 2023, by searching Pubmed, Embase, Web of Science, Cochrane Central Register of Controlled Trials, Cumulative Index of Nursing and Allied Health Literature, and three Chinese databases including CNKI, Wan Fang and VIP. In case of omittance, we also searched ClinicalTrials.gov. The search strategy was shown in supplemental Table 1.

Two reviewers independently conducted study screening based on the titles and abstracts, and further assessed the full texts of potential literature to identify eligible studies. Disagreements were resolved through discussion and consensus of the third reviewer.

Studies were selected based on the following inclusion criteria: (1) Peer-reviewed randomized controlled trials (RCTs) or comparable observational studies; (2) Studies compared low-dose IVIg (≤ 1 g/kg) and high-dose IVIg (˃ 1 g/kg); (3) Studies enrolled patients at age < 18 years; (4) Studies enrolled patients diagnosed with primary ITP, and the duration were less than 3 months [20]. Exclusion criteria were: (1) Duplicated studies. If multiple publications from the same study group occur, the one with the largest sample size or the most complete one was included; (2) Studies enrolled secondary ITP or previously treated patients; (3) The full texts were not available; (4) Studies published in languages other than Chinese and English.

### Data extraction

Two reviewers independently extracted data from each eligible study and then cross-checked the results. The following information was extracted: name of the first author, year of publication, type of studies, sample size, baseline characteristics (gender, age, PC before treatment), details of interventions (the dosage and course of IVIg, the type, dosage and course of glucocorticoid if combined with glucocorticoid), follow-up period. Disagreements between reviewers were resolved through discussion.

### Quality appraisal

The Risk of bias (ROB) tool recommended by the Cochrane Handbook for Systematic Reviews of Interventions was used to assess included RCTs [21]. The Newcastle-Ottawa Scale (NOS) was used to evaluate the methodological quality of included cohort studies [22]. Two reviewers independently performed the quality evaluation and obtained consensus through discussion.

### Data synthesis and statistical analysis

We used R statistical software (version 4.2.2) and Rstudio in this study. Meta-analysis was performed by using the “meta” package and the “metabin”, “metacont” and “metaprop” command. Relative risk (RR) was used for dichotomous variables, and mean difference (MD) or standard mean difference (SMD) was used for continuous variables as effect measure methods. The rates were calculated by pooling the reported proportion in each study. We used arcsin transformation if original proportion was not conformed to a normal distribution. We selected 95% Confidence Interval (CI) for interval estimation. Heterogeneity among included studies was tested by I² statistic [23]. If I² < 50%, the heterogeneity was considered low and the fixed-effects model was used for meta-analysis, while if I^2^ ≥ 50%, the heterogeneity was considered high and the random-effects model was used. The sources of heterogeneity were explored by subgroup analysis and sensitivity analysis. Subgroup analysis was performed based on different low-dose IVIg schemes, whether combined with intravenous glucocorticoid and the type of glucocorticoid. Sensitivity analysis was performed by removing one study from the analysis each time to evaluate the robustness of the pooled results. Funnel plot and Egger’s test were used to detect publication bias when a meta-analysis includes 10 or more studies [24, 25]. A two-sided *P* < 0.05 was considered statistically significant.

## Result

### Search results

**Fig. 1:**
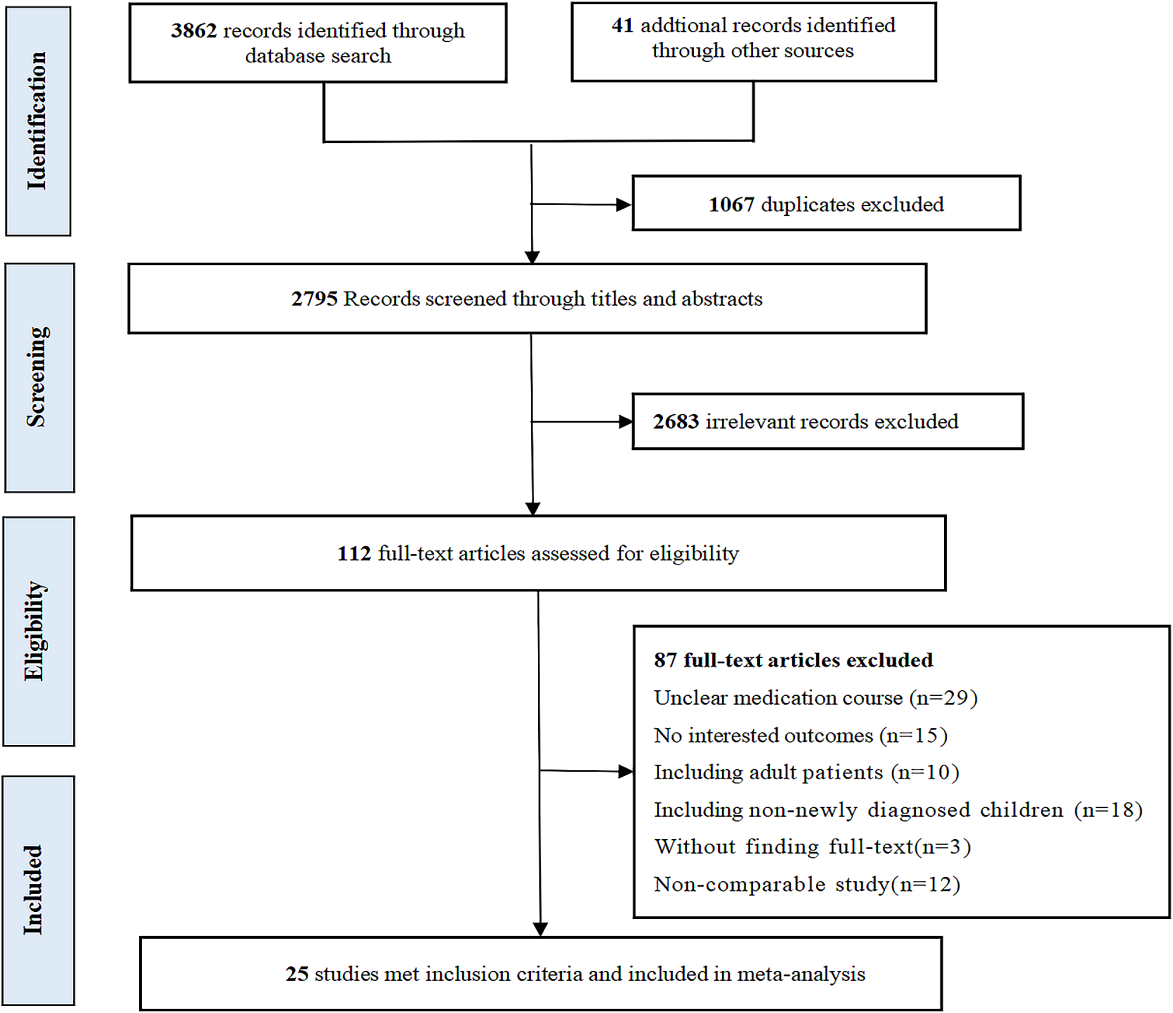
Flow diagram of literature search and selection

A total of 3903 articles were retrieved according to the above inclusion criteria. After removing 1067 duplicates, 2795 articles were screened the titles and abstracts and then 2683 irrelevant articles were excluded. The full texts of 112 potential articles were assessed and 25 eligible studies [26–50] were finally included in the systematic review and meta-analysis. Flow diagram summarizing the literature search and selection process is presented in Fig. 1.

### Study characteristics and quality assessment

The basic characteristics of included studies and patients were shown in Tables 1 and 2. A total of 1989 patients were included, with 991 patients in the LD-IVIg group and 998 patients in the HD-IVIg group. In 21 studies, the total doses of IVIg in the LD-IVIg group were 1 g/kg and 0.6 or 0.8 g/kg in the other 4 studies. While the total doses of IVIg in HD-IVIg group were mainly 2 g/kg. Twenty-two studies combined IVIg with glucocorticoid, most of which used dexamethasone (DXMS), and 3 studies used methylprednisolone (MP). The type, dose and course of glucocorticoid used in the LD-IVIg group and the HD-IVIg group were the same in each study.

**Table 1:** Basic information of included studies

Study ID	First author	Year of publication	Study design	Sample size(n)		Follow-up period	Outcomes
				LD-IVIg	HD-IVIg		
1	Bao H [26]	2017	RCT	44	44	3 months	ER; DR; AR
2	He WD [27]	2017	RCT	48	48	3 months	ER DR; AR Time of PC starting to rise; Time of PC rising to normal; Time of achieving hemostasis
3	Hou ZH [35]	2021	RCT	58	58	Not reported	ER
4	Ji LJ [29]	2022	RCT	46	46	Not reported	ER; AR Time of PC rising to normal Time of achieving hemostasis
5	Luo F [42]	2017	RCT	45	45	Not reported	ER Time of PC starting to rise; Time of achieving hemostasis
6	Su BX [44]	2016	RCT	38	40	3 months	ER; DR; AR Time of PC starting to rise; Time of PC rising to normal; Time of achieving hemostasis
7	Qin HZ [38]	2015	RCT	40	40	3 months	ER; DR; AR Time of PC starting to rise; Time of PC rising to normal; Time of achieving hemostasis
8	Yang YX [47]	2019	RCT	34	33	3 months	ER; DR; AR Time of achieving hemostasis
9	Yu ZJ [48]	2018	RCT	60	60	3 months	ER; DR Time of PC starting to rise; Time of PC rising to normal; Time of achieving hemostasis
10	Zhu W [50]	2014	RCT	65	65	Not reported	ER; AR
11	Zhao SL [49]	2018	RCT	17	17	6 months	ER; DR; AR Time of PC starting to rise; Time of PC rising to normal Time of achieving hemostasis
12	Feng L [30]	2019	RCT	34	34	3 months	ER; DR; AR Time of PC starting to rise; Time of PC rising to normal
13	He WH [34]	2019	RCT	31	31	3 months	ER; DR Time of PC starting to rise; Time of PC rising to normal
14	Shi L [43]	2019	RCT	32	32	Not reported	ER; AR Time of PC rising to normal; Time of achieving hemostasis
15	Liang CJ [37]	2017	RCT	28	26	Not reported	ER; AR Time of PC rising to normal; Time of achieving hemostasis
16	Tan YF [39]	2016	RCT	28	28	3 months	ER; DR Time of PC starting to rise; Time of PC rising to normal
17	Liu LK [40]	2003	RCT	45	45	Not reported	ER
18	Yang B [46]	2013	RCT	55	55	Not reported	ER; AR Time of PC rising to normal; Time of achieving hemostasis
19	Wang Y [45]	2022	RCT	19	19	Not reported	ER
20	Jin Y [36]	2020	RCT	25	25	Not reported	ER
21	Yang Y [32]	2022	RCT	51	51	Not reported	ER Time of PC starting to rise; Time of PC rising to normal; Time of achieving hemostasis
22	Hu XL [33]	2018	RCT	50	50	3 months	ER; DR
23	Gong CX [31]	2016	Cohort study	30	33	18 months	ER; DR
24	Liu YY [41]	2008	Cohort study	27	32	Not reported	ER
25	Huang HY [28]	2022	Cohort study	41	41	Not reported	ER; AR

*Abbreviation* RCT: randomized controlled trial; LD-IVIg: low-dose intravenous immunoglobulin; HD-IVIg: high-dose intravenous immunoglobulin; ER: effective rate; DR: durable remission rate; AR: adverse reaction rate; PC: platelet count

**Table 2:** Basic characteristics of included patients

Study	Gender F/M		Age (years)		PC before treatment (×10^9^)		Dose of IVIg (g/kg per day×days)		Type and dose of intravenous glucocorticoid (mg/kg per day×days)
	LD-IVIg	HD-IVIg	LD-IVIg	HD-IVIg	LD-IVIg	HD-IVIg	LD-IVIg	HD-IVIg	
Bao H [26]	21/23	19/25	5.94 ± 1.26	6.04 ± 1.31	13.64 ± 5.26	14.15 ± 5.19	0.2 × 5	0.4 × 5	DXMS (1.0 × 5)
He WD [27]	25/23	22/26	5.22 ± 1.13	5.51 ± 1.02	< 20 (17 patients) ≥ 20 (31 patients)	< 20 (14 patients) ≥ 20 (34 patients)	0.2 × 5	0.4 × 5	DXMS (1.5 × 5)
Hou ZH [35]	25/33	27/31	5.3 ± 1.5	5.1 ± 1.3	19.97 ± 5.11	20.15 ± 5.16	0.2 × 5	0.4 × 5	DXMS (0.5 × 5)
Ji LJ [29]	22/24	23/23	3.80 ± 1.25	3.85 ± 1.20	20.10 ± 5.03	20.12 ± 4.88	0.2 × 5	0.4 × 5	DXMS (1.5 × 5)
Luo F [42]	44/46		10.23 ± 3.13		< 20		0.2 × 5	0.4 × 5	DXMS (1.0 × 5)
Su BX [44]	20/18	20/20	6.2 ± 1.1	5.8 ± 1.2	17.2 ± 6.1	16.4 ± 5.9	0.2 × 5	0.4 × 5	DXMS (1.0 × 5)
Qin HZ [38]	Not reported		5.6 ± 1.7		< 25		0.2 × 5	0.4 × 5	DXMS (1.5 × 5)
Yang YX [47]	15/19	16/17	5.96 ± 1.08	5.90 ± 1.01	13.90 ± 5.23	13.78 ± 5.18	0.2 × 5	0.4 × 5	DXMS (1.0 × 5)
Yu ZJ [48]	27/33	26/34	5.4 ± 1.6	5.6 ± 1.2	< 10 (52 patients) 10–25 (68 patients)		0.2 × 5	0.4 × 5	DXMS (0.5 × 5)
Zhu W [50]	31/34	29/36	6.14 ± 2.47	6.21 ± 2.83	< 10 (27 patients) 10–25 (38 patients)	< 10 (30 patients) 10–25 (35 patients)	0.2 × 5	0.4 × 5	DXMS (0.5 × 5)
Zhao SL [49]	6/11	9/8	8.1 ± 2.1	7.7 ± 1.9	14.9 ± 4.5	14.6 ± 4.9	0.2 × 5	0.4 × 5	DXMS (0.5 × 5)
Feng L [30]	38/30		2–15		3–77		0.2 × 5	0.4 × 5	MP (20 × 3)
He WH [34]	10/21	11/20	9 months- 9 years	9 months- 10 years	Not reported		0.2 × 5	0.4 × 5	DXMS (0.5 × 5)
Shi L [43]	15/17	16/16	6.41 ± 1.26	6.65 ± 1.33	20.02 ± 5.13	19.95 ± 4.98	0.2 × 5	0.4 × 5	DXMS (0.5 × 7)
Liang CJ [37]	Not reported		3 months-14 years		22.65 ± 15.7	28.21 ± 18.82	0.2 × 5	0.4 × 5	DXMS (0.5 × 5)
Tan YF [39]	Not reported		5.6 ± 1.8		14–57		0.2 × 5	0.4 × 5	DXMS (1.0 × 5)
Liu LK [40]	44/46		7.35 ± 1.21		Not reported		0.2 × 5	0.4 × 5	MP (20 × 4)
Yang B [46]	26/29	28/27	6.80 ± 1.28	6.23 ± 1.22	19.98 ± 5.06	20.15 ± 5.08	0.4 × 2	1.0 × 2	No
Wang Y [45]	9/10	8/11	6.66 ± 1.28	6.78 ± 1.12	18.12 ± 2.44	18.08 ± 2.65	0.3 × 2	0.4 × 5	DXMS (0.5 × 7)
Jin Y [36]	8/17	9/16	2.82 ± 2.17	2.46 ± 1.86	< 20 × 10^9^/L		0.4 × 2	1.0 × 2	No
Yang Y [32]	30/21	22/29	3.9 ± 1.5	3.8 ± 1.3	Not reported		0.2 × 3	0.4 × 3	MP (20 × 4)
Hu XL [33]	12/38	11/39	4.21 ± 0.26	4.05 ± 0.12	Not reported		0.2 × 5	0.4 × 5	DXMS (1.0 × 5)
Gong CX [31]	18/12	18/15	2–16	3–16	1–19		0.2 × 5	0.4 × 5	No
Liu YY [41]	28/31		4.6(0.8-8.0)		6–21		1.0 × 1	0.4 × 5	DXMS (0.4 × 3)
Huang HY [28]	22/19	21/20	6.20 ± 2.46	6.24 ± 2.50	20.23 ± 5.26	20.14 ± 5.69	0.2 × 5	0.4 × 5	DXMS (1.0 × 5)

*Abbreviation* F: female; M: male; PC: platelet count; DXMS: dexamethasone; MP: methylprednisolone

Of the 25 eligible studies, there were 22 RCTs and 3 cohort studies. All of studies were single-center studies from China. The methodological quality of 3 cohort studies was moderate (Supplemental Table 2), and neither mentioned whether the confounding factors were controlled. We summarized the risk of bias assessment results of 22 RCTs in supplemental Fig. 1. In the domain of random sequence generation, 11 studies were low-risk, and the rest were unclear except Wang Y et al. [45] and He WH et al. [34]. In the domain of allocation concealment, only Liu LK et al. [40] was low-risk. Nearly all studies were lack of description of blinding in detail. In the remaining domains, the risk of bias in most trials was low.

### Comparison of effective rate between LD-IVIg and HD-IVIg treatment

**Fig. 2:**
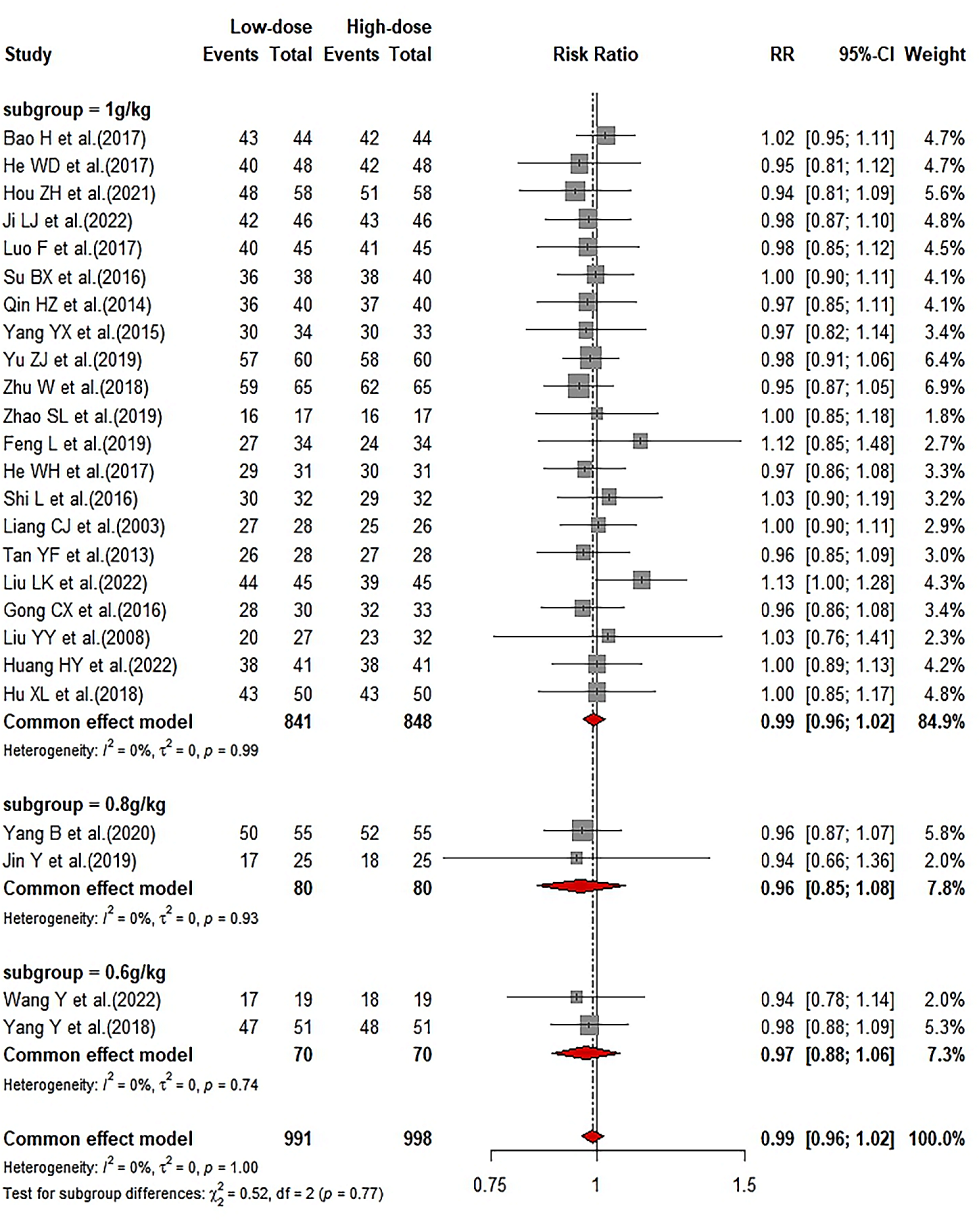
Forrest plot of the comparison of effective rate

All included studies reported the effective rate (ER). ER was defined as the proportion of patients who achieved either a complete (PC ≥ 100 × 10^9^/L) or partial (PC ≥ 50 × 10^9^/L) response. Meta-analysis results showed the pooled RR was 0.99 (95%CI: 0.96–1.02; I^2^ = 0), indicating no significant difference in ER between the LD-IVIg and the HD-IVIg groups (Fig. 2). After removing one study from the analysis each time, sensitivity analysis showed good consistency (Supplemental Fig. 2). Funnel plot (Supplemental Fig. 3) and Egger’s test (*P* = 0.8962) showed no significant publication bias.

In addition, we compared complete and partial response rate separately, and found no significant difference in complete response (RR: 0.97; 95%CI: 0.90–1.04; I^2^ = 0) and partial response rate (RR: 1.03; 95%CI: 0.91–1.18; I^2^ = 0) (Supplemental Fig. 4) between the two groups. Stable results of sensitivity analysis were shown in supplemental Fig. 5. Funnel plots (Supplemental Fig. 6) and Egger’s test (complete response rate: *P* = 0.2205; partial response rate: *P* = 0.0980) showed no publication bias.

### Effective rate of LD-IVIg and HD-IVIg treatment

**Fig. 3:**
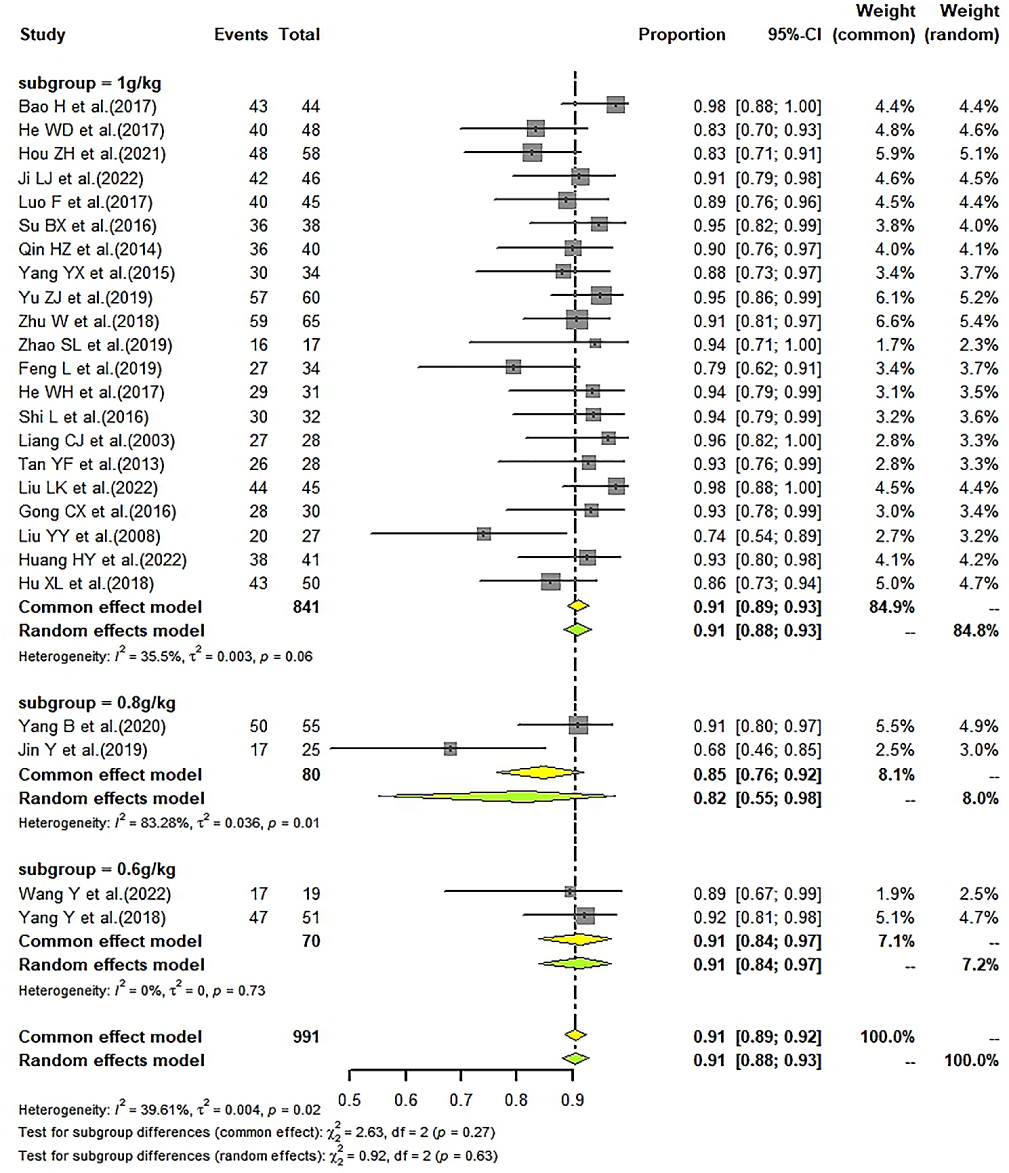
Forrest plot of the pooled effective rate in the LD-IVIg group

**Fig. 4:**
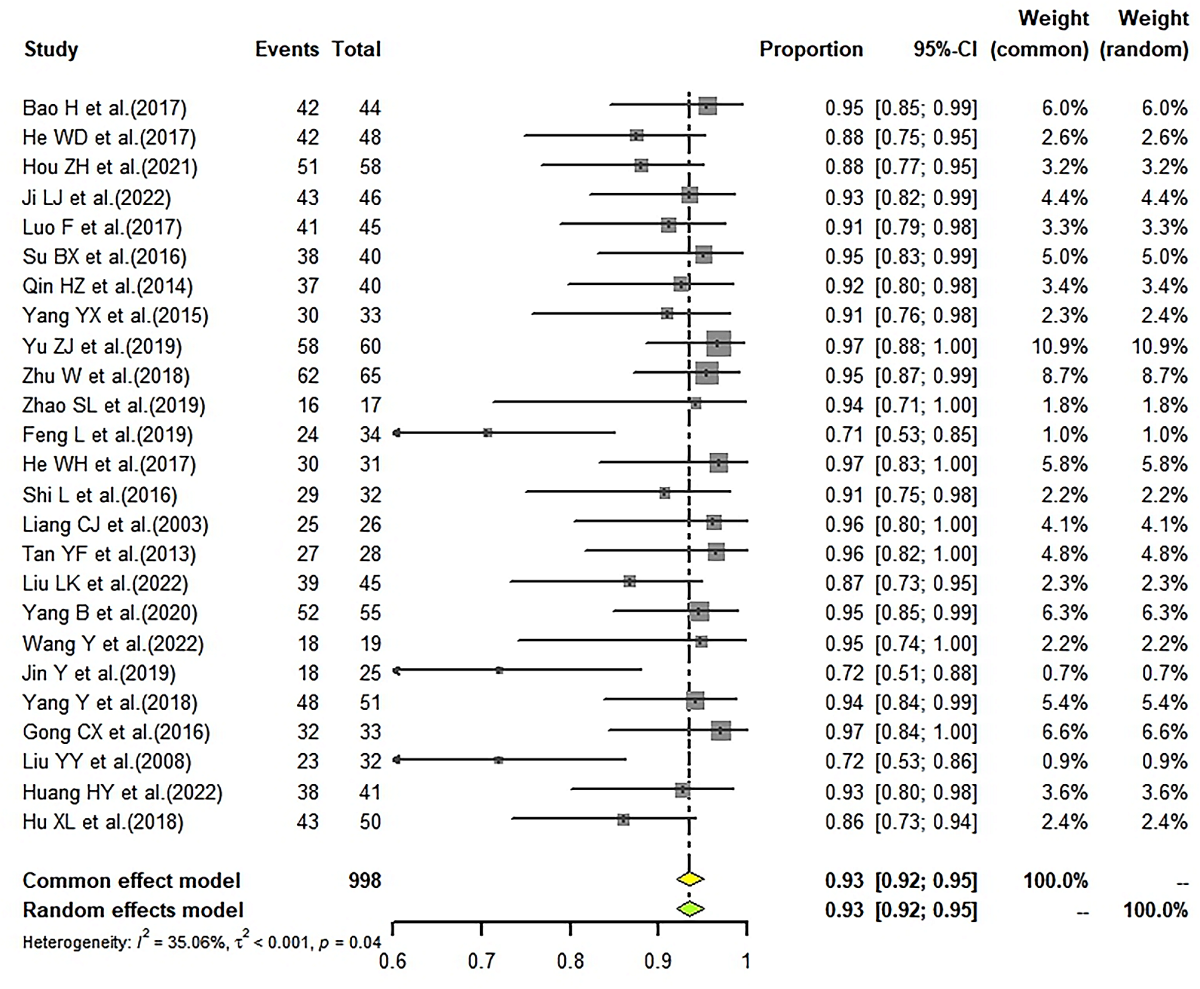
Forrest plot of the pooled effective rate in the HD-IVIg group

**Fig. 5:**
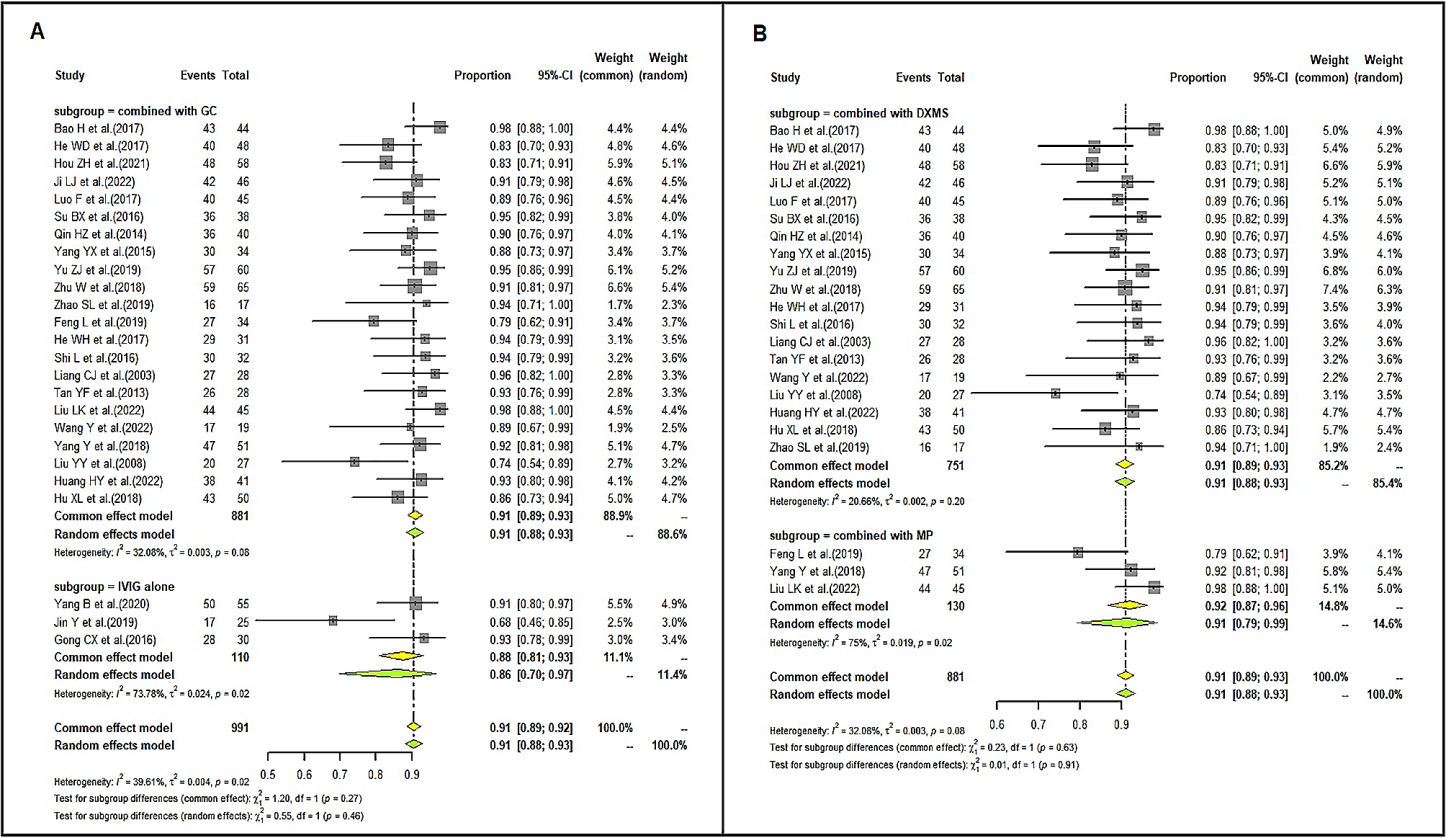
Subgroup analysis of whether combined with glucocorticoid (**A**) and the type of glucocorticoids (**B**). Abbreviation: GC: glucocorticoids; IVIG:intravenous immunoglobulin; DXMS: dexamethasone; MP: methylprednisolone

We found the pooled ER of the LD-IVIg group was 91% (95%CI: 89–92%; I^2^ = 39.61%) (Fig. 3), and that of the HD-IVIg group was 93% (95%CI: 92–95%; I^2^ = 35.06%) (Fig. 4). Subgroup analysis by different low-dose schemes showed the pooled ER was 91% (95%CI: 89–93%; I^2^ = 35.5%) in 1 g/kg, 82% (95%CI: 55–98%; I^2^ = 83.28%) in 0.8 g/kg and 91% (95%CI: 84–97%; I^2^ = 0%) in 0.6 g/kg subgroup. Combining with intravenous glucocorticoid was associated with a slightly higher ER (91% vs. 86%) (Fig. 5A). However, studies with different types of glucocorticoid showed similar ER (DXMS: 92% vs. MP: 91%) (Fig. 5B). Results of sensitivity analysis were stable (Supplemental Figs. 7 and 8). Funnel plots (Supplemental Figs. 9 and 10) and Egger’s test showed significant publication bias in the studies that reported ER of the HD-IVIg group (LD-IVIg group: *P* = 0.6287; HD-IVIg group: *P* < 0.0001).

Besides, the pooled complete response rate (60%) and partial response rate (29%) of the LD-IVIg group (Supplemental Fig. 11) were also close to those of the HD-IVIg group (complete response rate: 63%; partial response rate: 28%) (Supplemental Fig. 12). Subgroup analysis showed the pooled complete and partial response rate were respectively 61% and 29% in 1 g/kg, 50% and 28% in 0.8 g/kg, 59% and 30% in 0.6 g/kg subgroup. Results of sensitivity analysis were shown in supplemental Figs. 13 and 14. Funnel plots (Supplemental Figs. 15 and 16) and results of Egger’s test (complete response rate of the LD-IVIg group: *P* = 0.5234; partial response rate of the LD-IVIg group: *P* = 0.2412; complete response rate of the HD-IVIg group: *P* = 0.2170; partial response rate of the HD-IVIg group: *P* = 0.0544) showed no publication bias.

### Comparison of durable remission rate between LD-IVIg and HD-IVIg treatment

**Fig. 6:**
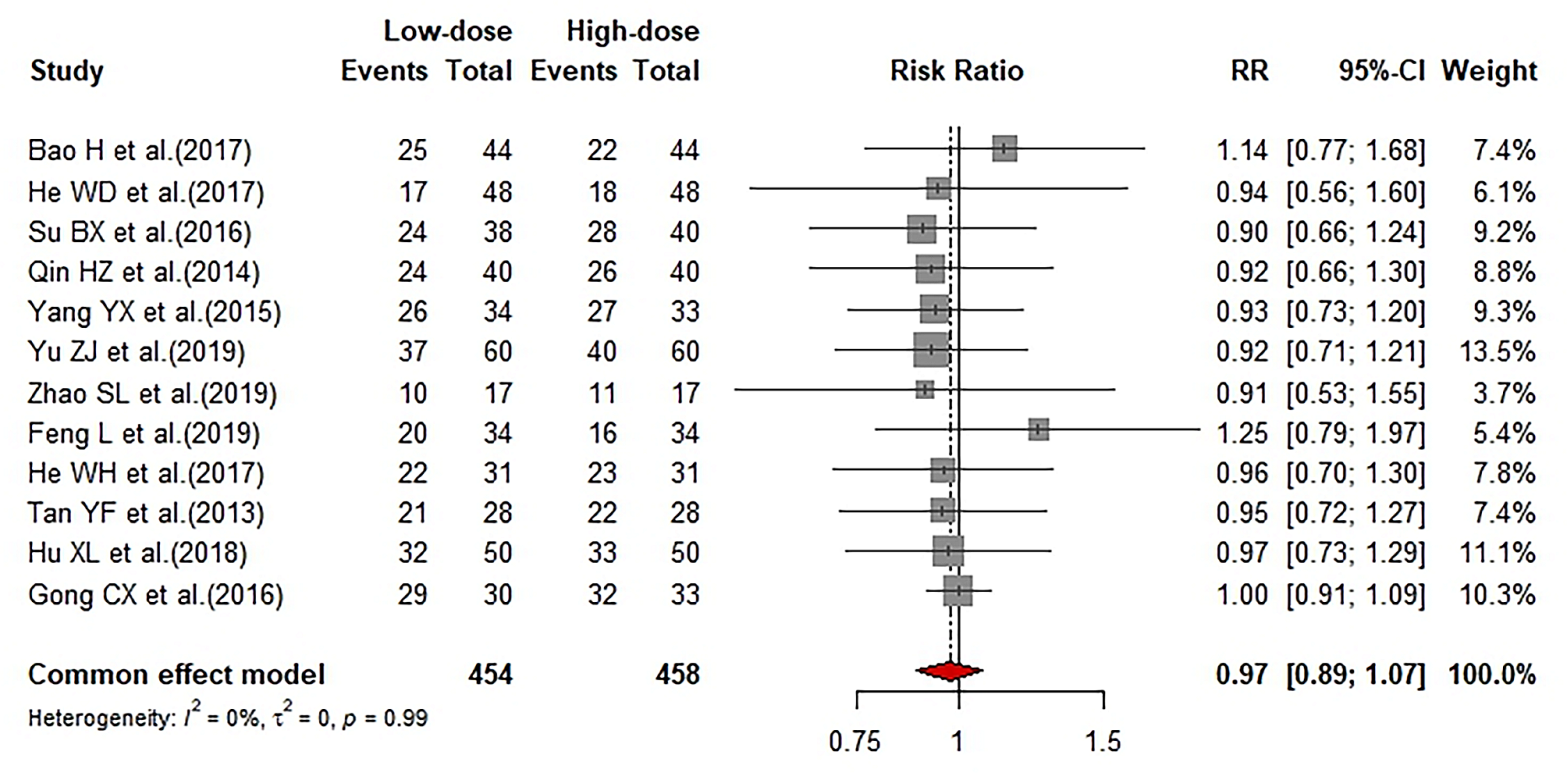
Forrest plot of comparison of durable remission rate

Twelve studies reported durable remission rate (DR). Durable remission was defined as patient’s PC remaining within the normal range for at least 3 months without recurrence and medical treatment. All studies used 1 g/kg IVIg in the LD-IVIg group. The follow-up period was mostly 3 months and up to 18 months. Meta-analysis results showed there was no significant difference between the two groups (RR: 0.97; 95%CI: 0.89–1.07; I^2^ = 0) (Fig. 6). Sensitivity analysis revealed robust results (Supplemental Fig. 17). Funnel plot (Supplemental Fig. 18) and Egger’s test (*P* = 0.6842) showed no publication bias.

### Durable remission rate of LD-IVIg and HD-IVIg treatment

**Fig. 7:**
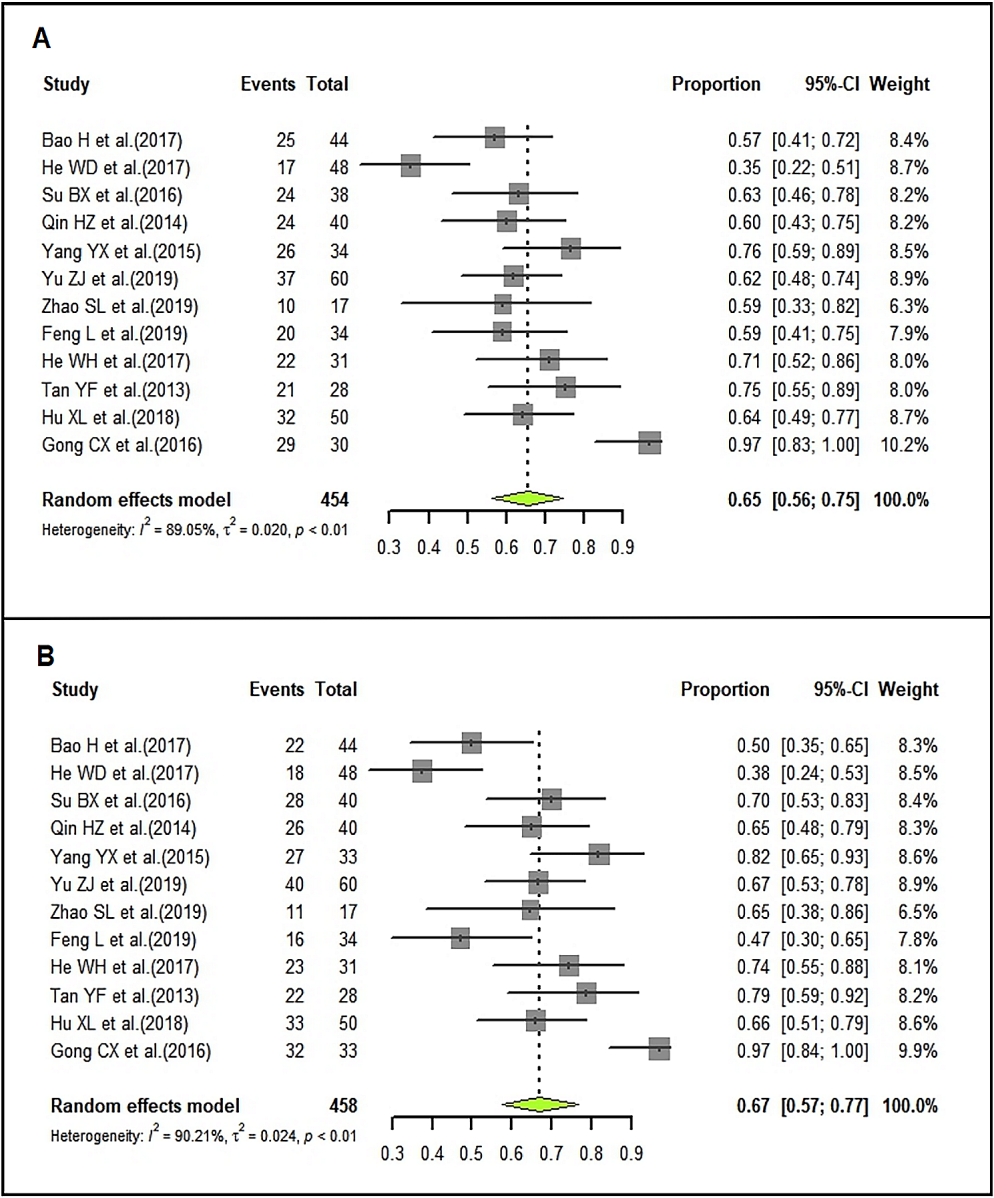
Forrest plots of the pooled durable remission rate in the LD-IVIg group (**A**) and the HD-IVIg group (**B**)

In LD-IVIg group, the pooled DR rate was 65% (95%CI: 56–75%; I^2^ = 89.05%) (Fig. 7A), and that of HD-IVIg group was 67% (95%CI: 57–77%; I^2^ = 90.21%) (Fig. 7B). Sensitivity analysis showed a slight change across the included studies (Supplemental Fig. 19). Funnel plots were shown in supplemental Fig. 20. Egger’s test showed there was publication bias (LD-IVIg group: *P* = 0.0032; HD-IVIg group: *P* = 0.0022) in the 12 studies.

### Comparison of the time of pc starting to rise between LD-IVIg and HD-IVIg treatment

**Fig. 8:**
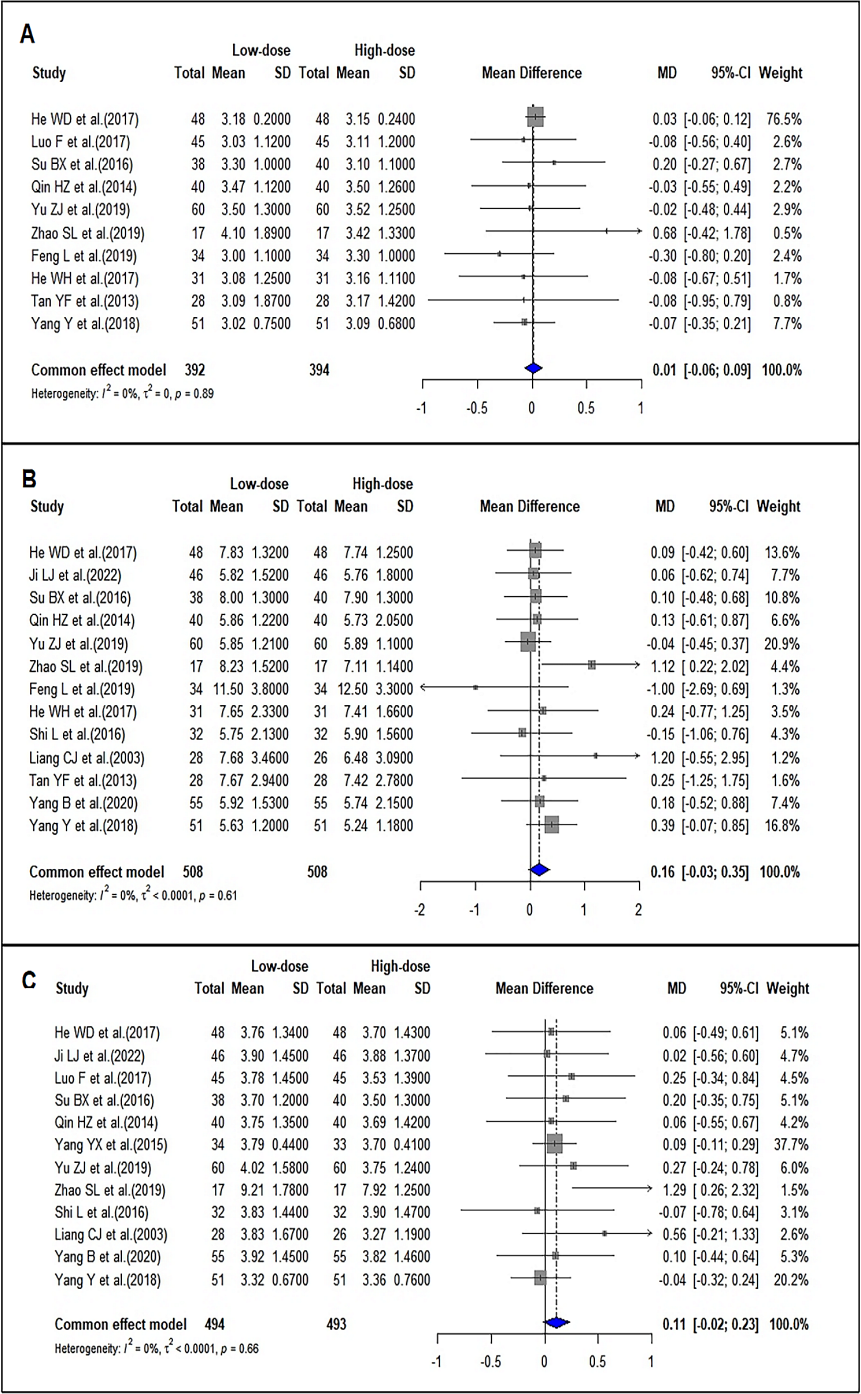
Forrest plots of comparison of the time of platelet count starting to rise (**A**), rising to normal (**B**), and achieving hemostasis (**C**)

Ten studies reported the time of PC starting to rise (d). The pooled MD was 0.01 (95%CI: -0.06–0.09; *I*^*2*^ = 0), suggesting no significant difference between the two groups (Fig. 8A). Sensitivity analysis found similar results (Supplemental Fig. 21A). Funnel plot and the results of Egger’s test (*P* = 0.6727) suggested that there was no significant publication bias (Supplemental Fig. 22A).

### Comparison of the time of pc rising to normal between LD-IVIg and HD-IVIg treatment

Thirteen studies reported the time of PC returning to normal (d). The pooled MD was 0.16 (95%CI: -0.03–0.35; I^2^ = 0), suggesting no significant difference between the two groups (Fig. 8B). Sensitivity analysis showed the results changed slightly (Supplemental Fig. 21B). Funnel plot (Supplemental Fig. 22B) and the results of Egger’s test (*P* = 0.6372) suggested that there was no significant publication bias.

### Comparison of the time of achieving hemostasis between LD-IVIg and HD-IVIg treatment

Twelve studies reported the time of hemorrhage stopping (d). The pooled MD was 0.11 (95%CI: -0.02–0.23; I^2^ = 0), suggesting no significant difference between the two groups (Fig. 8C). Sensitivity analysis showed the results were stable. (Supplemental Fig. 21C). Funnel plot (Supplemental Fig. 22C) and the results of Egger’s test (*P* = 0.0679) suggested that there was no significant publication bias.

### Comparison of adverse reaction rate between LD-IVIg and HD-IVIg treatment

**Fig. 9:**
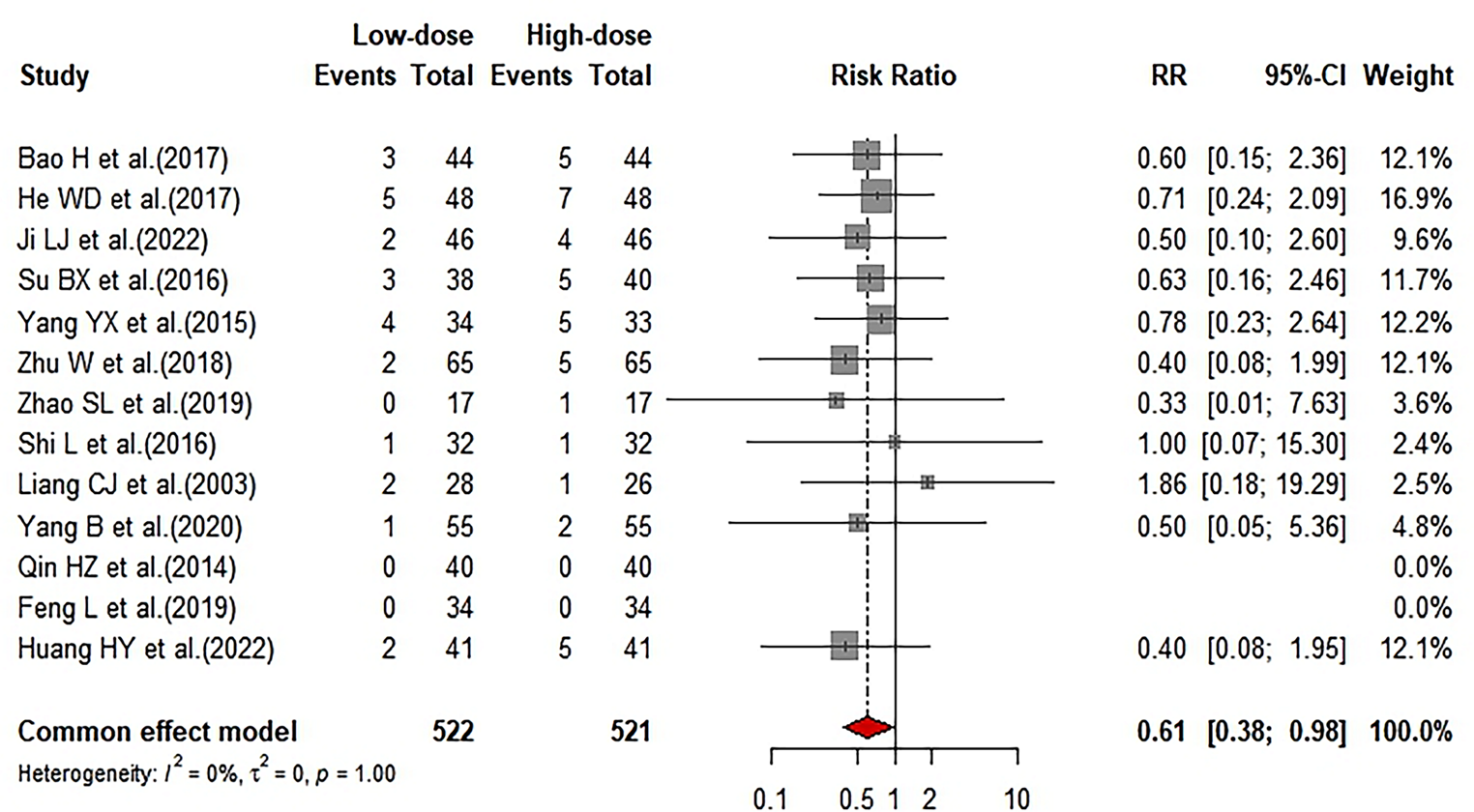
Forrest plot of comparison of adverse reaction rate

A total of 13 studies reported adverse reactions. Meta-analysis results showed adverse reaction rate (AR) of the LD-IVIg group was significantly lower than that of the HD-IVIg group (RR: 0.61; 95%CI: 0.38–0.98; I^2^ = 0) (Fig. 9). Sensitivity analysis showed the results of the 13 studies were similar (Supplemental Fig. 23A). No publication bias was found by observing the funnel plot (Supplemental Fig. 23B) and Egger’s test (*P* = 0.9170).

### Adverse reaction rate of LD-IVIg and HD-IVIg treatment

**Fig. 10:**
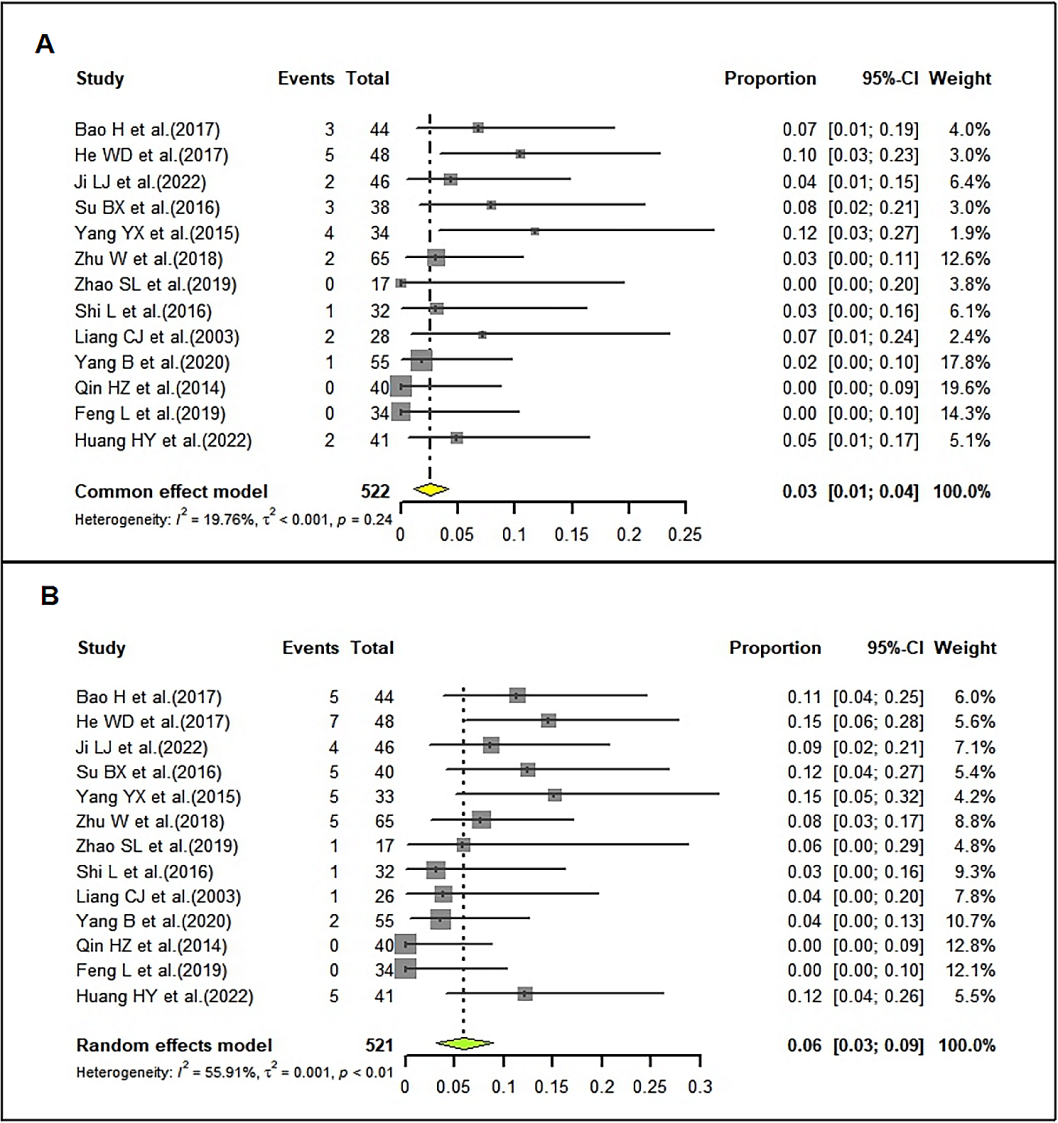
Forrest plots of the pooled adverse reaction rate in the LD-IVIg group (**A**) and the HD-IVIg group (**B**)

The pooled AR rate of LD-IVIg group was 3% (95%CI: 1–4%; I^2^ = 19.76%) (Fig. 10A), which was markedly lower than that of HD-IVIg group (6%; 95%CI: 3–9%; I^2^ = 55.91%) (Fig. 10B). The results of sensitivity analysis were shown in supplemental Fig. 24. Funnel plots (Supplemental Fig. 25) and Egger’s test (LD-IVIg group: *P* = 0.0004; HD-IVIg group: *P* < 0.0001) found publication bias across the 13 studies.

In addition, the most common adverse reaction reported in the included studies was fever (42%). Other reactions such as flushing (11%), skin rash (7%), phlebitis (8%), allergy (17%), headache and dizziness (8%), nausea and vomiting (9%) occurred much less frequently.

## Discussion

In this study, we included 25 clinical studies compared different dosages of IVIg, involving 1989 newly diagnosed ITP children from China. Our systematic review and meta-analysis investigated 3 main questions: (1) Whether low-dose IVIg (≤ 1 g/kg) is as effective as conventional high-dose IVIg (2 g/kg)? (2) How effective are those different low-dose schemes? (3) Does the combination therapy further improve the therapeutic effect than IVIg alone?

Regarding the first question, our results showed the pooled ER including the complete and partial response rate were similar between the low-dose IVIg (≤ 1 g/kg) and high-dose IVIg (2 g/kg) groups, and there were also no significant differences in the pooled DR and the time of PC increase and hemorrhage improvement, suggesting reduced-dose IVIg was equally effective and can be used as an initial treatment.

In order to identify the efficacy of different low-dose regimens, we performed a proportion meta-analysis. We found that the pooled ER of 0.6 g/kg subgroup was equal to 1 g/kg subgroup (91%) but higher than 0.8 g/kg subgroup (82%). This may be due to the fact that all studies using 0.6 g/kg also combined with glucocorticoid, while all studies using 0.8 g only gave IVIg. Since all the studies reported DR used 1 g/kg IVIg (65%), we were unable to perform a subgroup analysis of other schemes.

Regarding the third question, we further conducted a subgroup analysis of whether glucocorticoid was combined and found combining with intravenous glucocorticoid was associated with a higher ER when compared with using IVIg only (91% vs. 86%). So, this confirmed that a higher ER in 0.6 g/kg subgroup was related to combination therapy. Several studies have shown that glucocorticoids can reduce the formation of autoantibody and binding to antigens, inhibit the phagocytic effect of monocyte-macrophage system on platelets, and stimulate medullary hematopoiesis function and platelet maturation [51, 52]. The combination of glucocorticoid and IVIg can raise PC faster than IVIg alone [53, 54]. Based on our subgroup analysis, we also found the pooled ER of combining with DXMS was close to that of MP, indicating the type of glucocorticoid had little influence on the effect.

As for safety, Kato et al.16 enrolled 748 patients treated with IVIg for different diseases and found adverse events were recorded in 8.5% of patients received higher doses of IVIg while only 0.8% of patients received lower doses experienced adverse events [17]. Our results showed the pooled AR of the HD-IVIg group was nearly double the LD-IVIg group, indicating the risks may be dose-related and mostly associated with high-dose administration.

Furthermore, IVIg-associated adverse events were mainly divided into immediate and delayed adverse reactions [55]. The most common immediate adverse reactions were mild influenza-like symptoms such as fever, flush, headache, fatigue and dizziness [56]. Delayed adverse reactions were severe and rare, such as thrombosis, aseptic meningitis, hemolysis, renal failure, and nervous system diseases [57]. In our study, most of reported adverse reactions were mild, which could be alleviated by slowing down the infusion speed, discontinuing infusion or symptomatic treatment.

Although a systematic review and meta-analysis comparing 1 g/kg and 2 g/kg has been already published in 2010 (OR: 1.00, 95%CI: 0.61–1.63) [58], some apparent flaws such as insufficient literature search and incomplete evaluation of the effect of low-dose IVIg existed. In this study, we have performed a comprehensive search, pooled the effective rate, and performed the subgroup analysis based on different low-dose schemes. Our results may provide more information for selecting an appropriate dosage of IVIg, which may ultimately lead to a reduction in medical costs.

However, there are some limitations in this current study. First, all the studies were from China. Most of the included RCTs did not describe the blinding and allocation concealment methods in detail and the potential confounding factors were not controlled in the cohort studies, which limited the quality of our results. Second, only a few studies used 0.6 or 0.8 g/kg IVIg and the sample sizes were small, which might lead to uncertain estimation of their efficacy. Third, there was a lack of long-term follow-up in the included studies, so we failed to assess whether low-dose IVIg can reduce the likelihood of developing chronic ITP.

In conclusion, we identified the efficacy of 1 g/kg IVIg was equal to 2 g/kg, and even 0.6 or 0.8 g/kg was also effective. A combination with glucocorticoids can improve therapeutic effects, so we suggested combining with glucocorticoids when giving low-dose IVIg. In the future, more high-quality studies with appropriate sample sizes are needed to identify the efficacy of IVIg less than 1 g/kg and explore the effect of improving prognosis.

### Electronic supplementary material

Below is the link to the electronic supplementary material.

Supplementary Material 1

Supplementary Material 2

## Data Availability

Any data and material in this manuscript are available within the article and its supplementary materials. If someone wants to request the data please contact Xiangge Ren (Email: 13223881563@sohu.com).
