# Supplementary material for: Can low-dose intravenous immunoglobulin be an alternative to high-dose intravenous immunoglobulin in the treatment of children with newly diagnosed immune thrombocytopenia: a systematic review and meta-analysis
Source: BMC Pediatr. 2024 Mar 21;24:199. doi: 10.1186/s12887-024-04677-3 (PMC10956331; doi:10.1186/s12887-024-04677-3)
Supplement: Supplementary file 1 — Supplementary Material 1 [file 12887_2024_4677_MOESM1_ESM.docx]

**Supplemental table 1:** Search strategy

| Search | Query |
| --- | --- |
| #1 | "Purpura, Thrombocytopenic, Idiopathic"[MESH Terms] OR "Immune Thrombocytopenic Purpura"[Title/Abstract] OR "Immune Thrombocytopenia"[Title/Abstract] OR "Autoimmune Thrombocytopenia"[Title/Abstract] OR "Autoimmune Thrombocytopenic Purpura"[Title/Abstract] OR "ITP"[Title/Abstract] |
| #2 | " Immunoglobulin, Intravenous"[MESH Terms] OR "Immunoglobulin G"[Title/Abstract] OR "IVIG"[Title/Abstract] OR "IVIg"[Title/Abstract] |
| #3 | “child*"[MESH Terms] OR "adolescent"[MESH Terms] OR "teen"[Title/Abstract] OR "toddler"[Title/Abstract] OR "pediatric*"[Title/Abstract] |
| #4 | #1 AND #2 AND #3 |

**Supplemental table 2:** **NOS assessment results of cohort studies**

| **Study name:** **Gong CX et al.** | |
| --- | --- |
| **Items** | **Response options** |
| Selection | |
| *Representativeness of the exposed cohort？* | *** |
| *Selection of the non exposed cohort* | *** |
| *Ascertainment of exposure* | *** |
| *Demonstration that outcome of interest was not present at start of study* | no |
| Comparability | |
| *Comparability of cohorts on the basis of the design or analysis* | * |
| Outcome | |
| *Assessment of outcome* | no description |
| *Was follow-up long enough for outcomes to occur* | * |
| *Adequacy of follow up of cohorts* | *** |
| ***Score*** | **6** |

| **Study name: Liu YY et al.** | |
| --- | --- |
| **Items** | **Response options** |
| Selection | |
| *Representativeness of the exposed cohort？* | *** |
| *Selection of the non exposed cohort* | *** |
| *Ascertainment of exposure* | *** |
| *Demonstration that outcome of interest was not present at start of study* | no |
| Comparability | |
| *Comparability of cohorts on the basis of the design or analysis* | * |
| Outcome | |
| *Assessment of outcome* | no description |
| *Was follow-up long enough for outcomes to occur* | no |
| *Adequacy of follow up of cohorts* | no description |
| ***Score*** | **4** |

| **Study name:** **Huang HY et al.** | |
| --- | --- |
| **Items** | **Response options** |
| Selection | |
| *Representativeness of the exposed cohort？* | *** |
| *Selection of the non exposed cohort* | *** |
| *Ascertainment of exposure* | *** |
| *Demonstration that outcome of interest was not present at start of study* | no |
| Comparability | |
| *Comparability of cohorts on the basis of the design or analysis* | * |
| Outcome | |
| *Assessment of outcome* | no description |
| *Was follow-up long enough for outcomes to occur* | no |
| *Adequacy of follow up of cohorts* | no description |
| ***Score*** | **4** |
