# Supplementary material for: Can low-dose intravenous immunoglobulin be an alternative to high-dose intravenous immunoglobulin in the treatment of children with newly diagnosed immune thrombocytopenia: a systematic review and meta-analysis
Source: BMC Pediatr. 2024 Mar 21;24:199. doi: 10.1186/s12887-024-04677-3 (PMC10956331; doi:10.1186/s12887-024-04677-3)
Supplement: Supplementary file 2 — Supplementary Material 2 [file 12887_2024_4677_MOESM2_ESM.docx]

**Supplemental figure 1:** Risk of bias assessment results of RCTs

**

Note:** 1: Bao H et al.; 2: He WD et al.; 3: Hou ZH et al.; 4: Ji LJ et al.; 5: Luo F et al.; 6: Su BX et al.;7: Qin HZ et al.; 8: Yang YX et al.; 9: Yu ZJ et al.; 10: Zhu W et al.; 11: Zhao SL et al.; 12: Feng L et al.; 13: He WH et al.; 14: Shi L et al.; 15: Tan YF et al.; 16: Liu LK et al.; 17: Yang B et al.; 18: Wang Y et al.; 19: Jin Y et al.; 20: Yang Y et al.; 21: Hu XL et al.; 22: Liang CJ et al.

**Supplemental figure 2:** Comparison of effective rate between LD-IVIg and HD-IVIg treatment: Sensitivity analysis


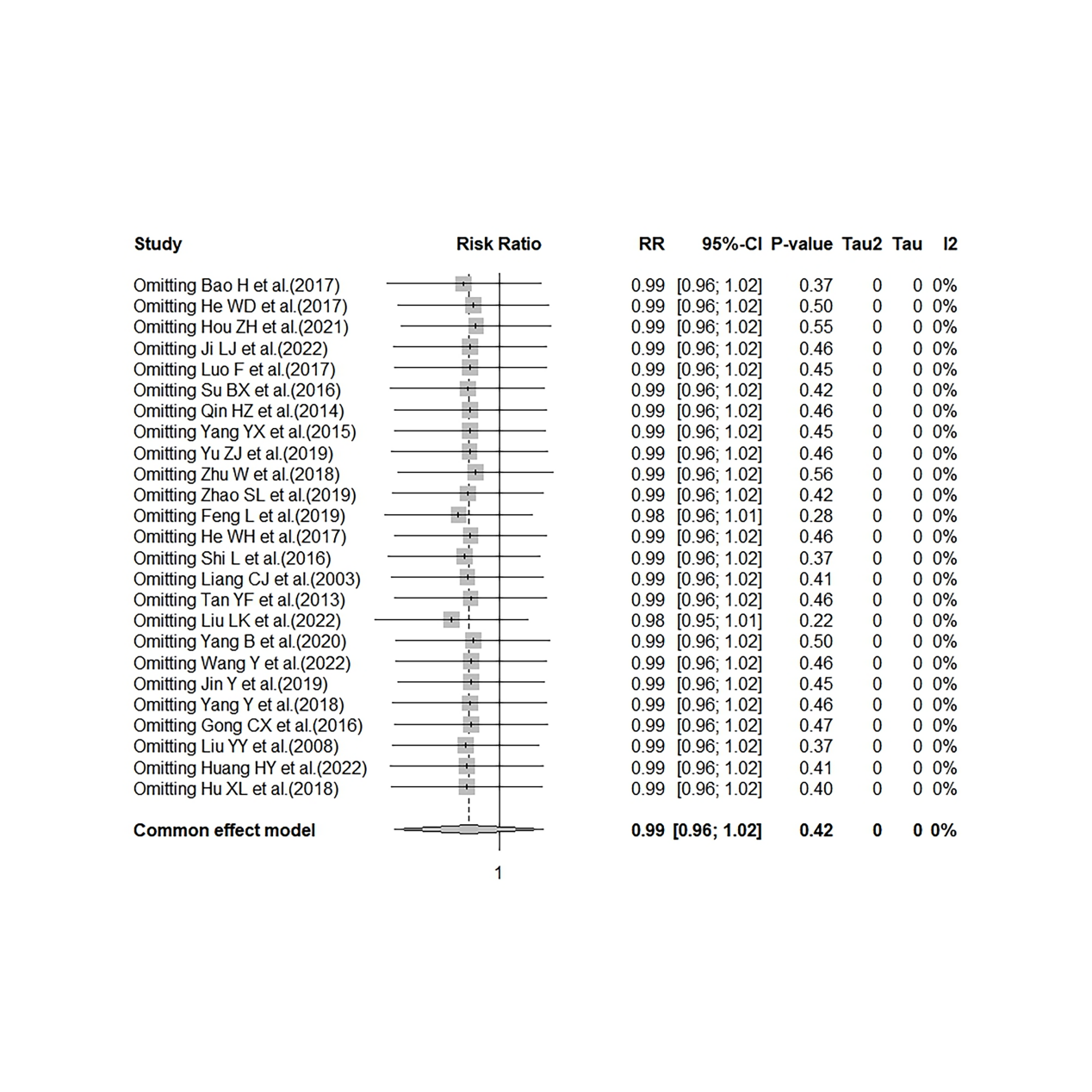


**Supplemental figure 3:** Comparison of effective rate between LD-IVIg and HD-IVIg treatment: Funnel plot





**Supplemental figure 4:** Forrest plots of the comparison of complete response rate (A) and partial response rate (B)


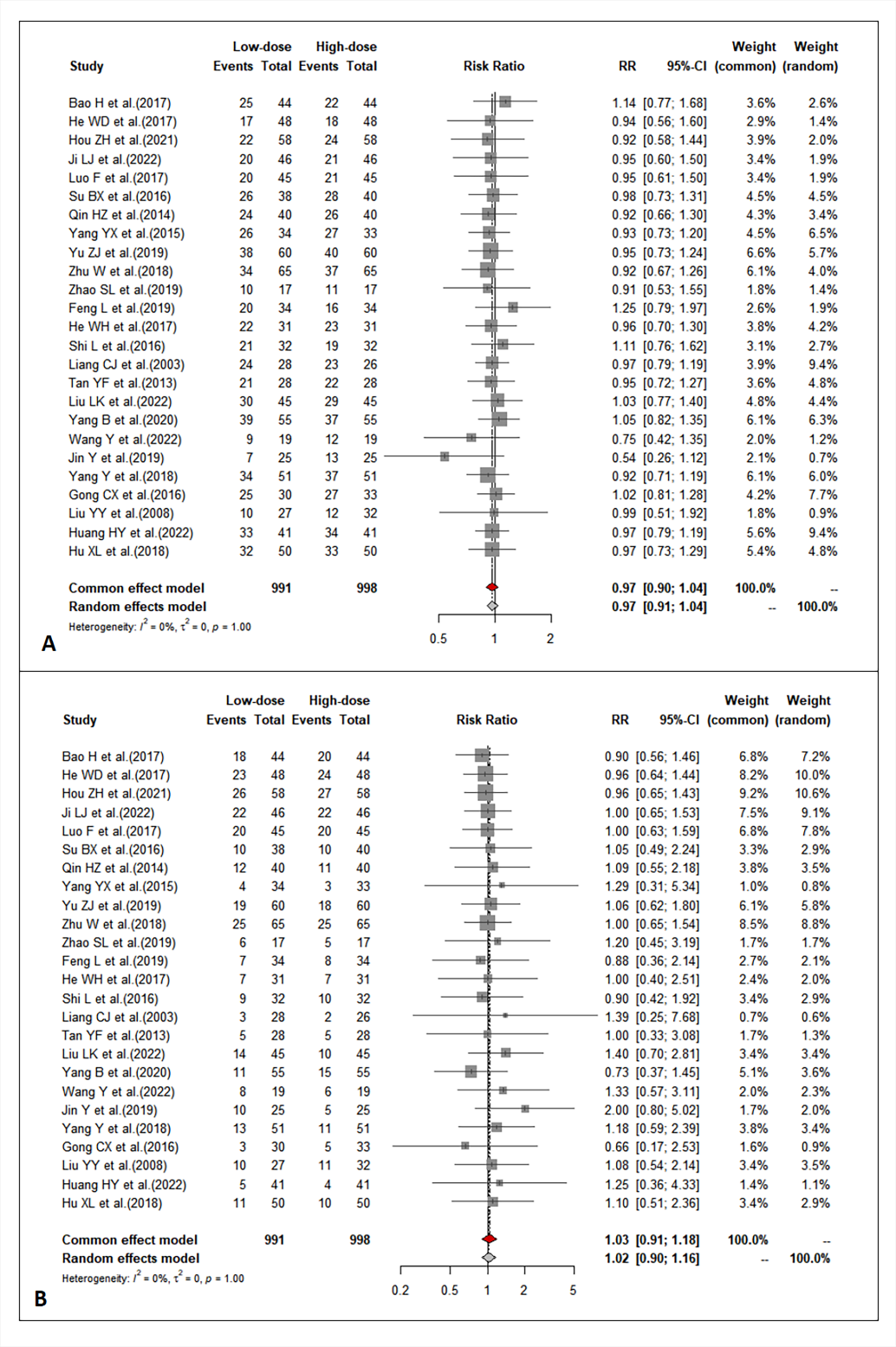


**Supplemental figure 5:** Comparison of complete response rate (A) and partial response rate (B) between LD-IVIg and HD-IVIg treatment: Sensitivity analysis

**
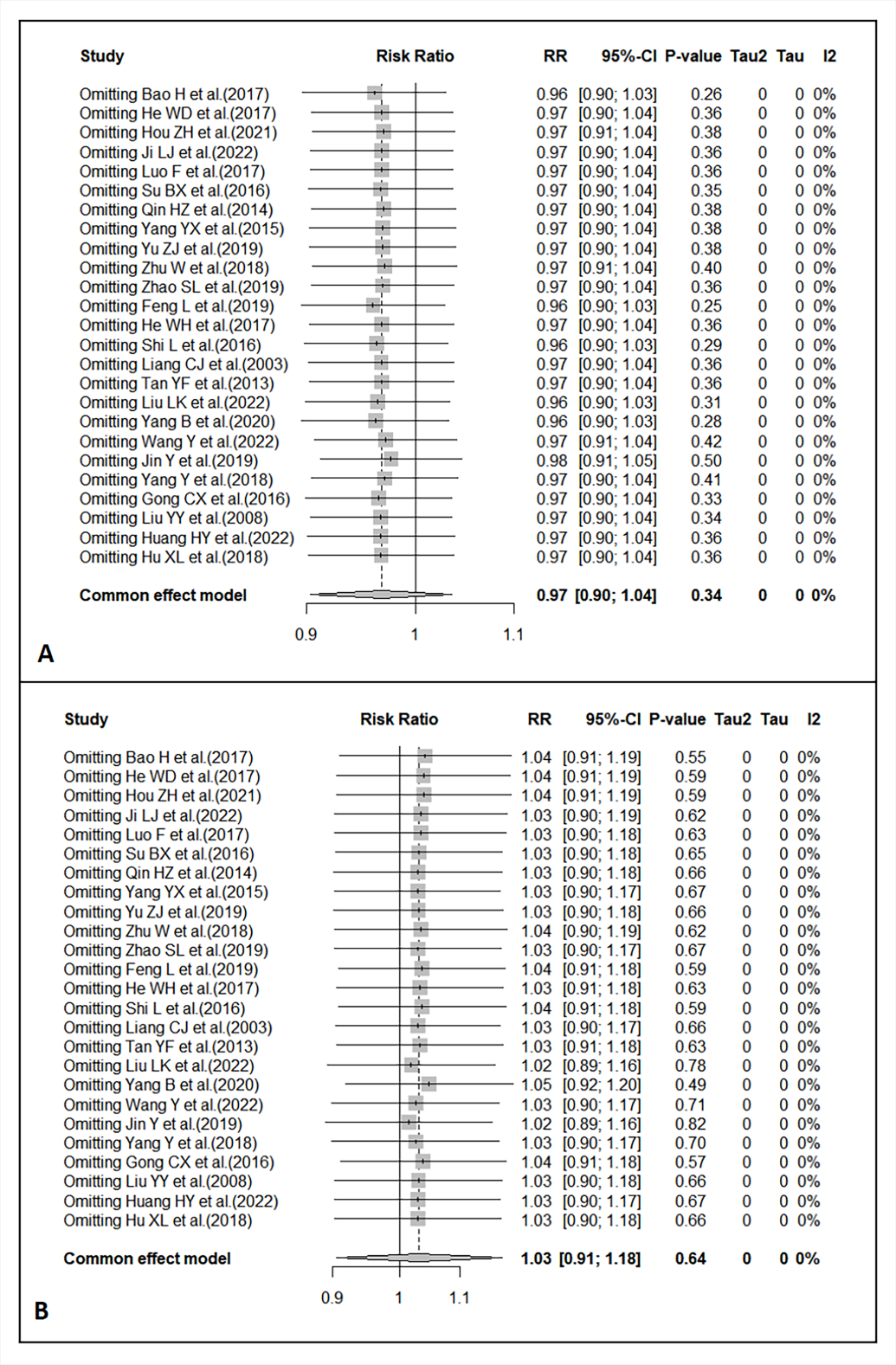
**

**Supplemental figure 6:** Comparison of complete response rate (A) and partial response rate (B) between LD-IVIg and HD-IVIg treatment: Funnel plots

**
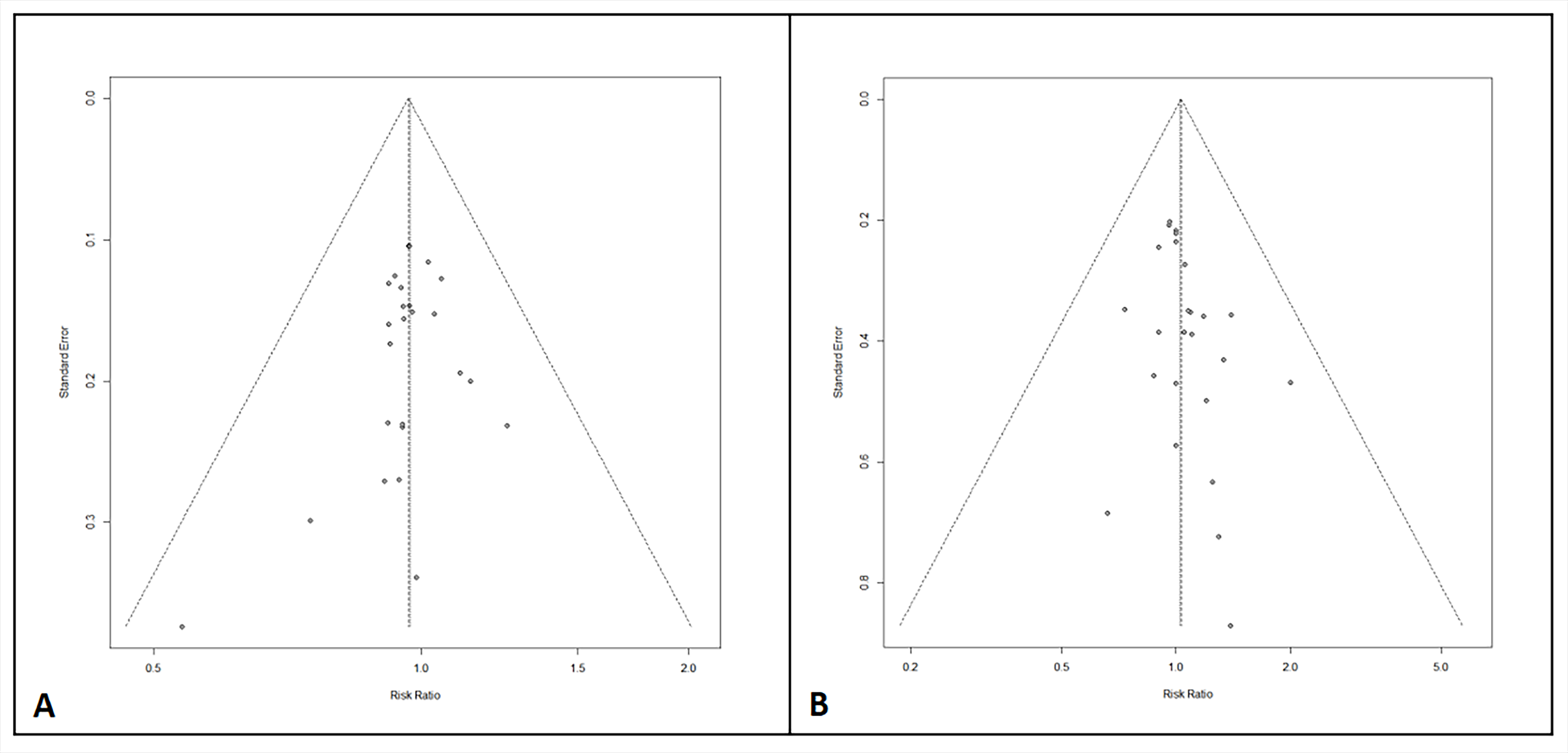
**

**Supplemental figure 7:** The pooled effective rate of LD-IVIg treatment: Sensitivity analysis


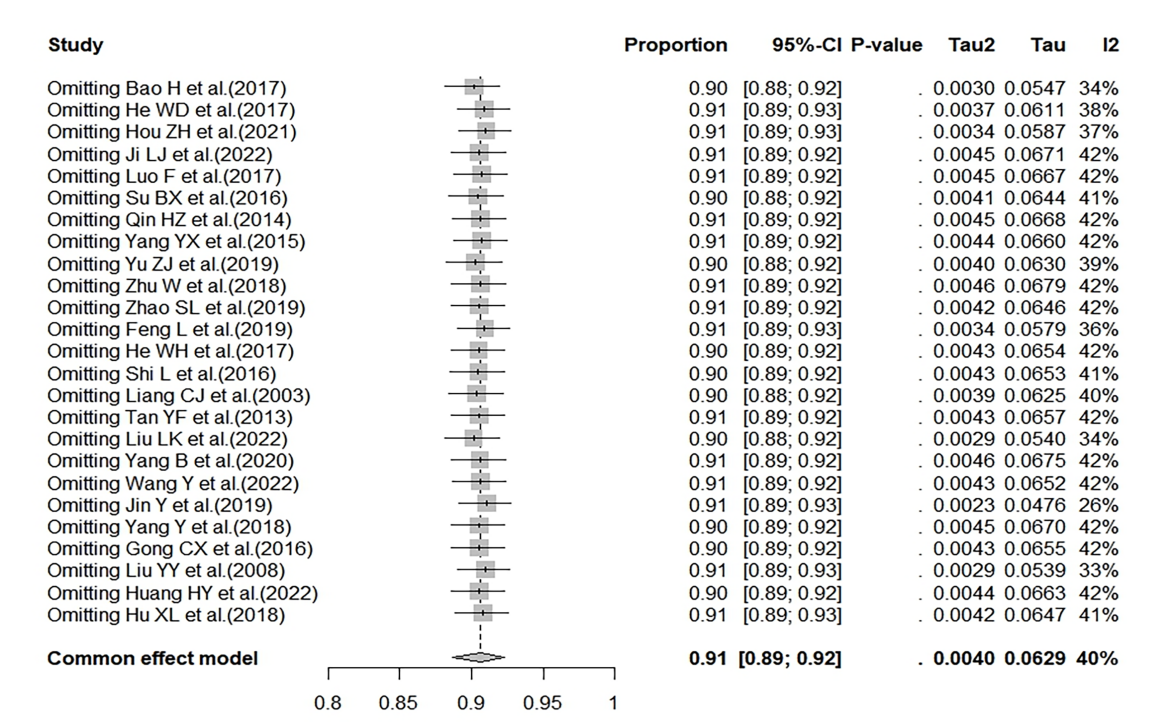


**Supplemental figure 8:** The pooled effective rate of HD-IVIg treatment: Sensitivity analysis


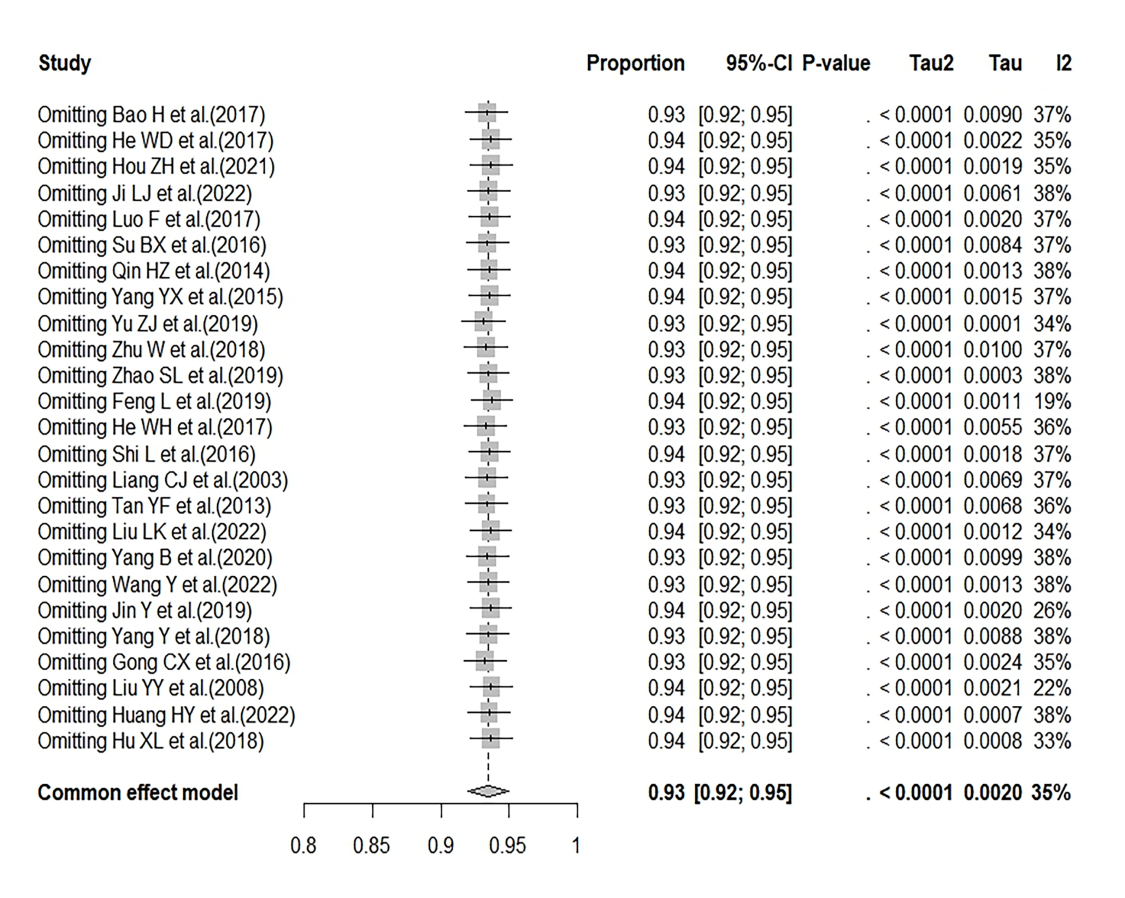


**Supplemental figure 9:** The pooled effective rate of LD-IVIg treatment: Funnel plot





**Supplemental figure 10:** The pooled effective rate of HD-IVIg treatment: Funnel plot





**Supplemental figure 11:** Forrest plots of the pooled complete response rate (A) and partial response rate (B) of LD-IVIg treatment

**
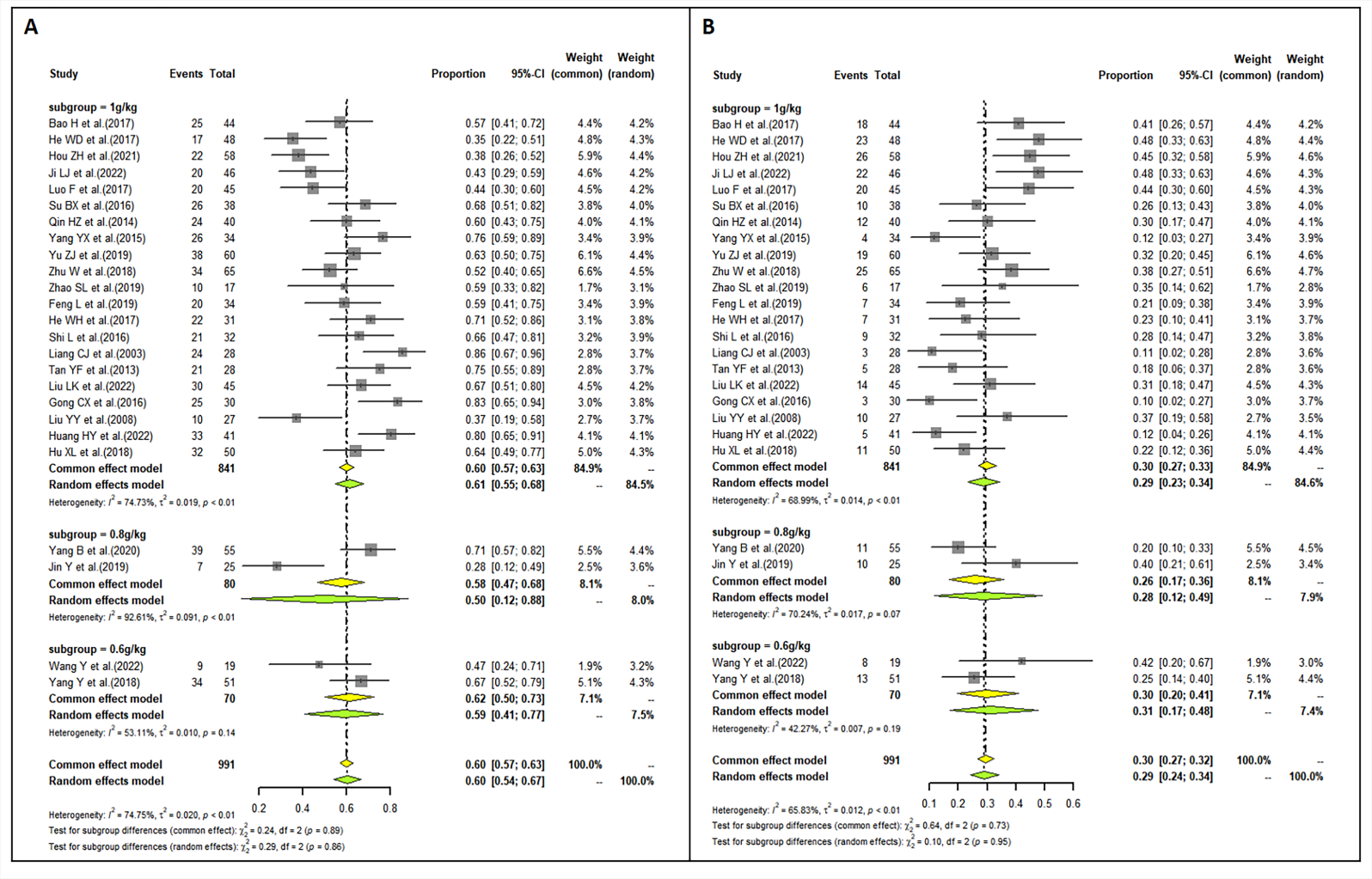
**

**Supplemental figure 12:** Forrest plots of the pooled complete response rate (A) and partial response rate (B) of HD-IVIg treatment


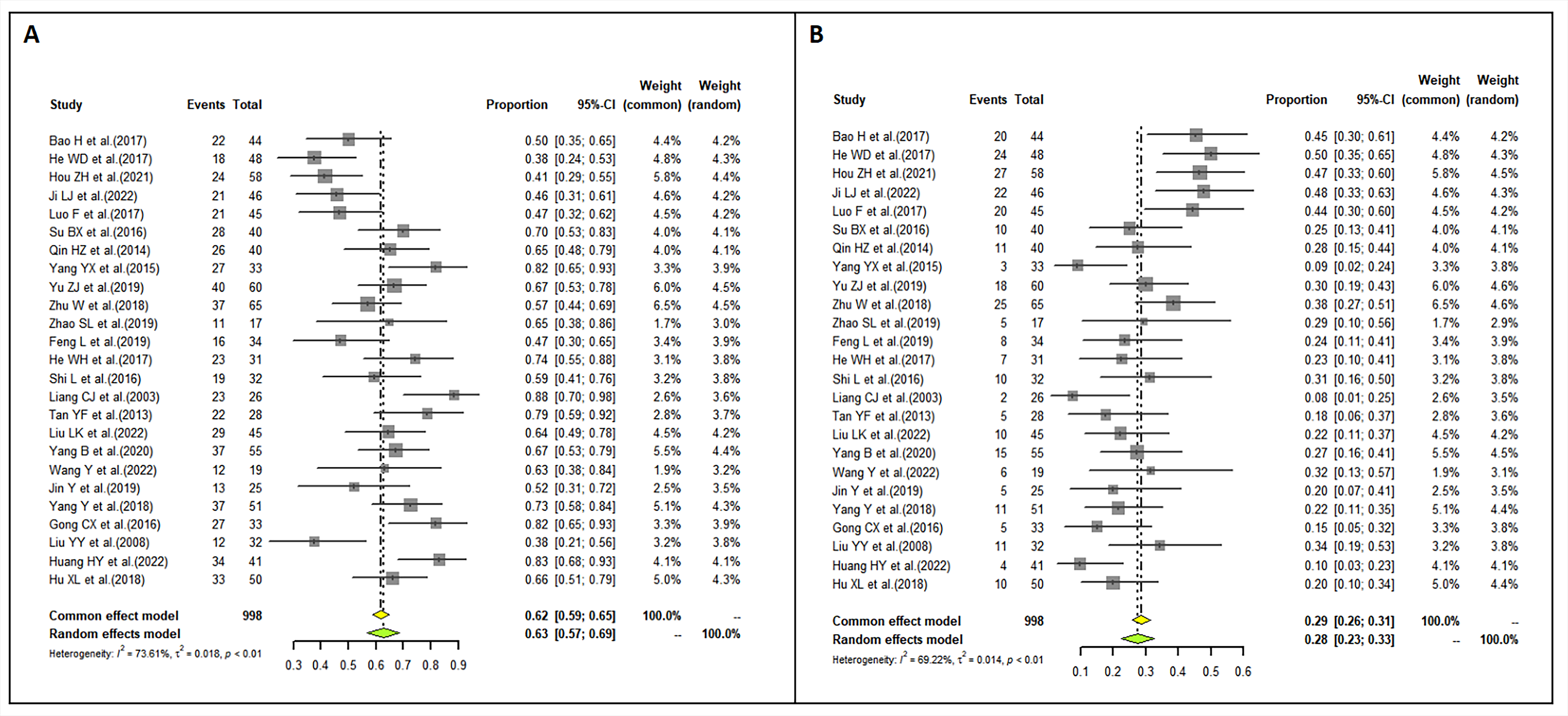


**Supplemental figure 13:** The pooled complete response rate (A) and partial response rate (B) of LD-IVIg treatment: Sensitivity analysis

**Supplemental figure 14:** The pooled complete response rate (A) and partial response rate (B) of HD-IVIg treatment: Sensitivity analysis


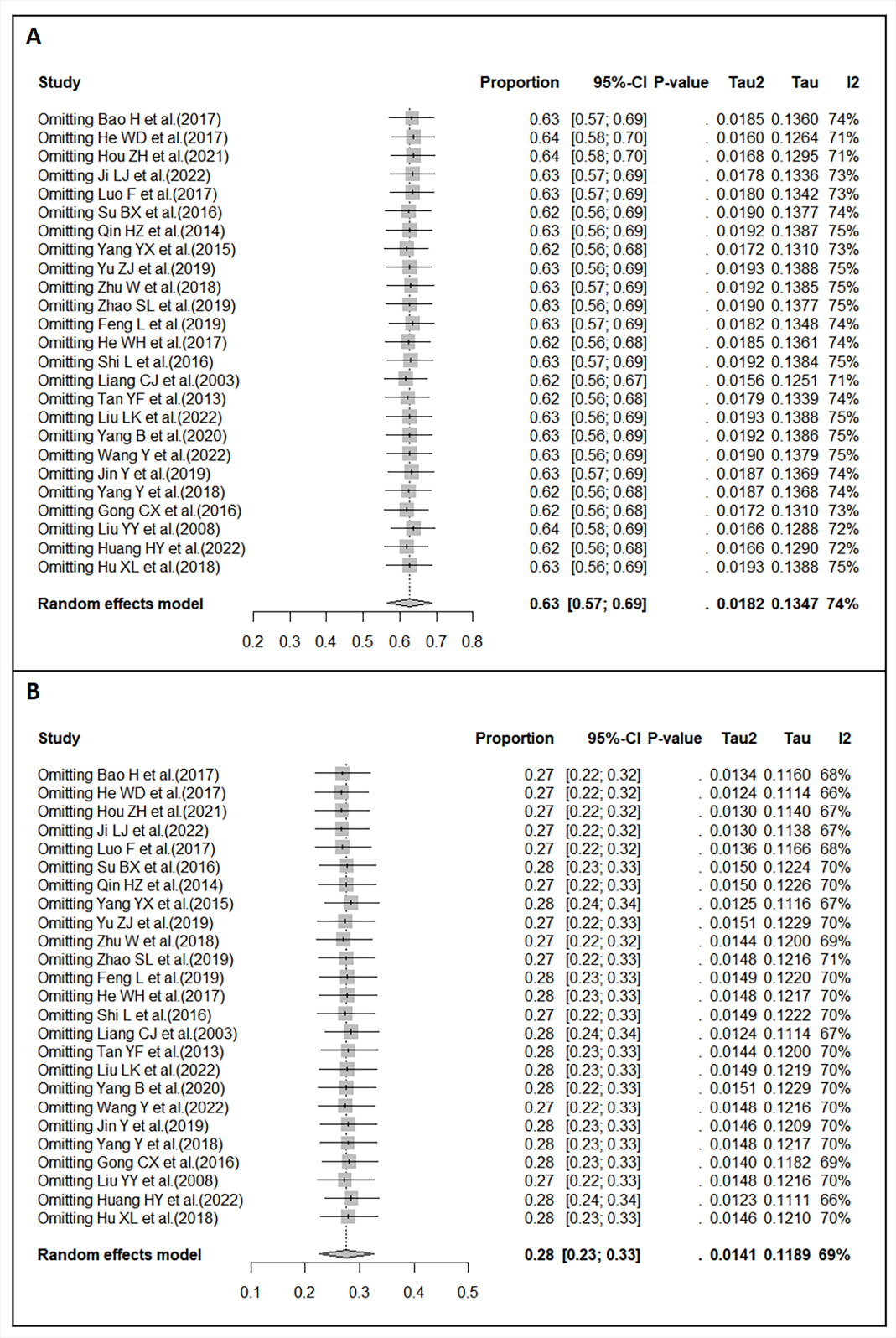


**Supplemental figure 15:** The pooled complete response rate (A) and partial response rate (B) of LD-IVIg treatment: Funnel plots


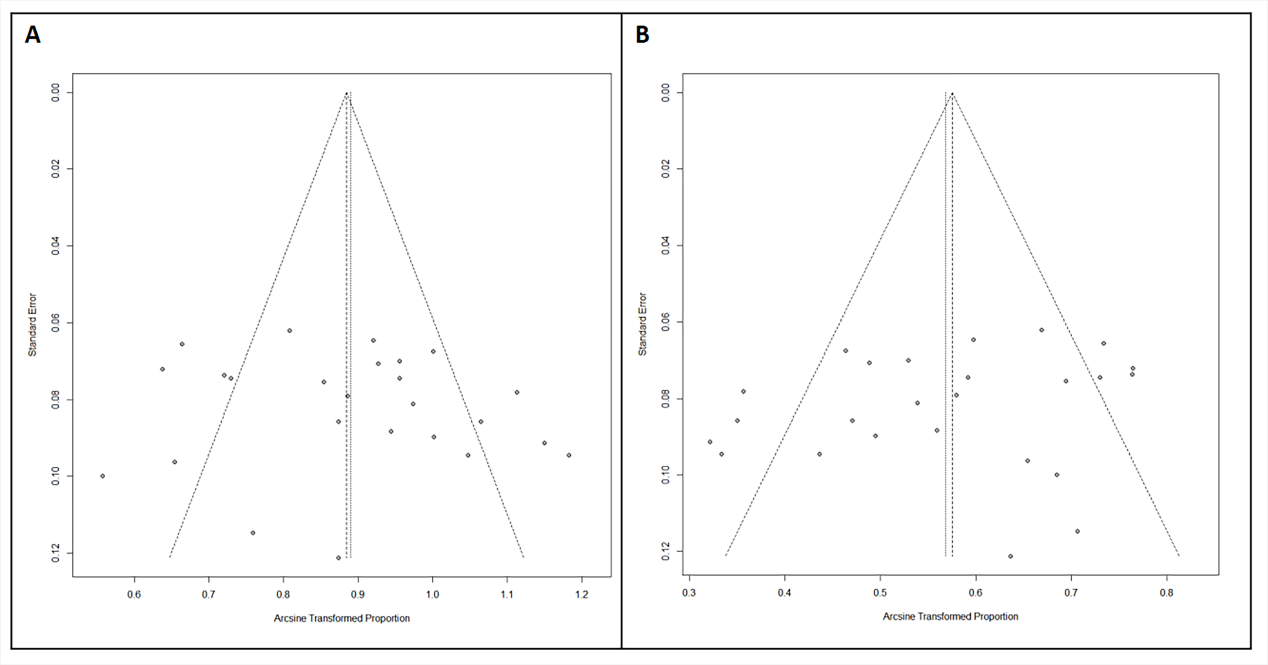


**Supplemental figure 16** The pooled complete response rate (A) and partial response rate (B) of HD-IVIg treatment: Funnel plots


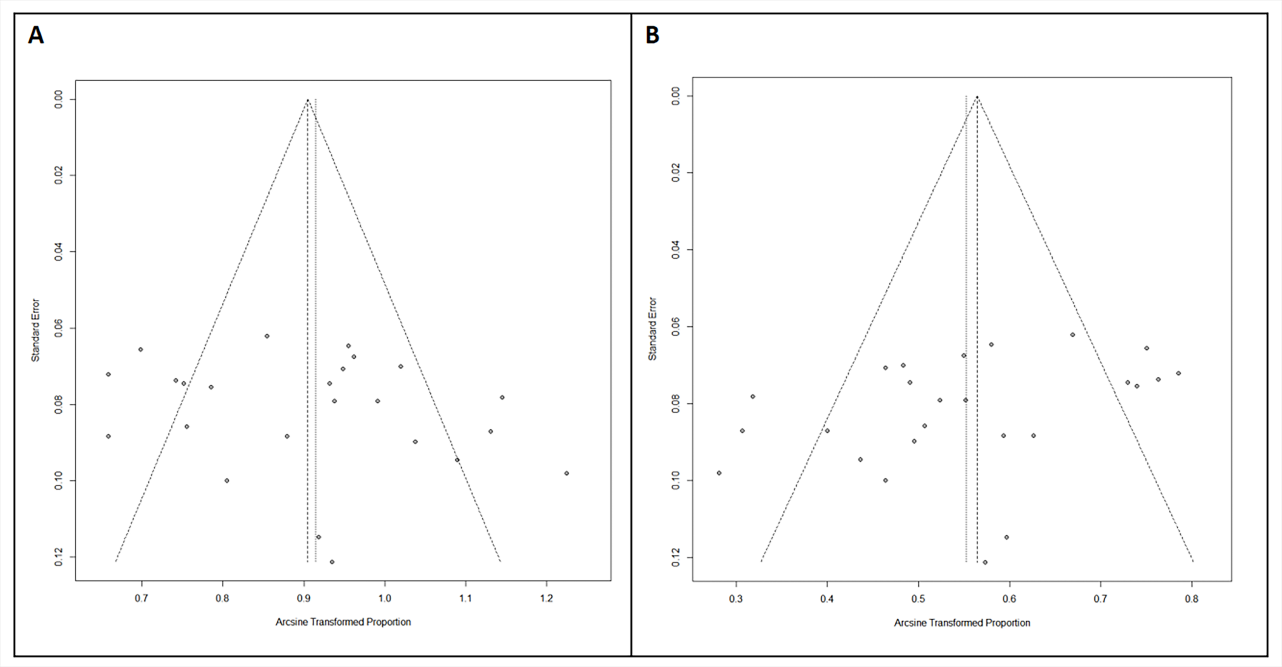


**Supplemental figure 17:** Comparison of durable remission rate between LD-IVIg and HD-IVIg treatment: Sensitivity analysis





**Supplemental figure 18:** Comparison of durable remission rate between LD-IVIg and HD-IVIg treatment: Funnel plot





**Supplemental figure 19:** The pooled durable remission rate: Sensitivity analysis of LD-IVIg treatment (A) and HD-IVIg treatment (B)





**Supplemental figure 20:** The pooled durable remission rate: Funnel plots

of LD-IVIg treatment (A) and HD-IVIg treatment (B)





**Supplemental figure 21:** Sensitivity analysis of comparison of the time of platelet count starting to rise (A), rising to normal (B), and achieving hemostasis(C) between LD-IVIg and HD-IVIg treatment

**

**

**Supplemental figure 22:** Funnel plots of comparison of the time of platelet count starting to rise (A), rising to normal (B), and achieving hemostasis (C) between LD-IVIg and HD-IVIg treatment





**Supplemental figure 23:** Sensitivity analysis (A) and funnel plot (B) of comparison of the adverse reaction rate between LD-IVIg and HD-IVIg treatment


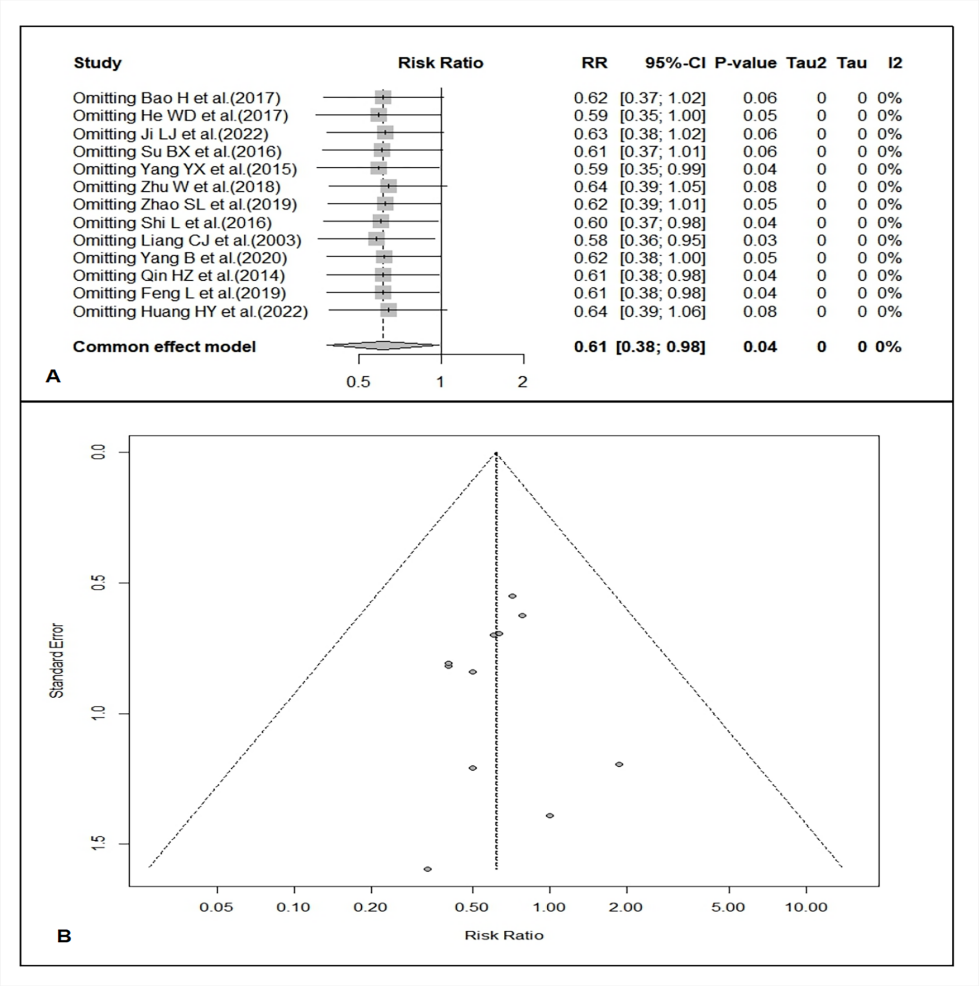


**Supplemental figure 24:** The pooled adverse reaction rate: Sensitivity analysis of LD-IVIg treatment (A) and HD-IVIg treatment (B)


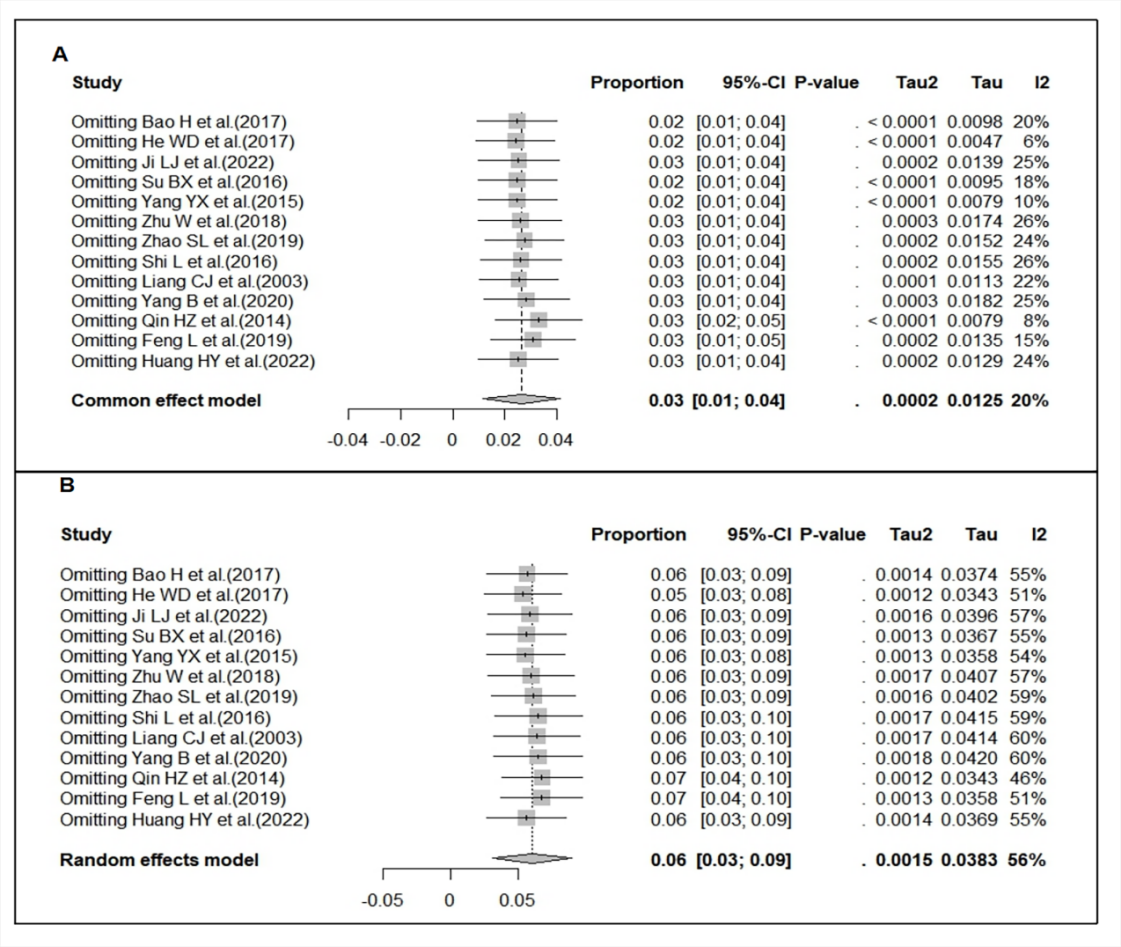


**Supplemental figure 25:** The pooled adverse reaction rate: Funnel plots

of LD-IVIg treatment (A) and HD-IVIg treatment (B)
